# ABA signalling promotes cell totipotency in the shoot apex of germinating embryos

**DOI:** 10.1093/jxb/erab306

**Published:** 2021-06-27

**Authors:** Baojian Chen, Martijn Fiers, Bas J W Dekkers, Lena Maas, G Wilma van Esse, Gerco C Angenent, Yang Zhao, Kim Boutilier

**Affiliations:** 1 Bioscience, Wageningen University and Research, AA Wageningen, Netherlands; 2 Laboratory for Molecular Biology, Wageningen University and Research, AP, Wageningen, Netherlands; 3 Wageningen Seed Lab, Laboratory for Plant Physiology, Wageningen University and Research Centre, AA, Netherlands; 4 Shanghai Center for Plant Stress Biology, and CAS Center of Excellence in Molecular Plant Sciences, Chinese Academy of Sciences, Shanghai, China; 5 University of Maryland, USA

**Keywords:** ABA, Arabidopsis, 2,4-D, mature zygotic embryo, somatic embryogenesis, seed maturation, totipotency

## Abstract

Somatic embryogenesis (SE) is a type of induced cell totipotency where embryos develop from vegetative tissues of the plant instead of from gamete fusion after fertilization. SE can be induced *in vitro* by exposing explants to growth regulators, such as the auxinic herbicide 2,4-dichlorophenoxyacetic acid (2,4-D). The plant hormone abscisic acid (ABA) has been proposed to be a downstream signalling component at the intersection between 2,4-D- and stress-induced SE, but it is not known how these pathways interact to induce cell totipotency. Here we show that 2,4-D-induced SE from the shoot apex of germinating *Arabidopsis thaliana* seeds is characterized by transcriptional maintenance of an ABA-dependent seed maturation pathway. Molecular–genetic analysis of Arabidopsis mutants revealed a role for ABA in promoting SE at three different levels: ABA biosynthesis, ABA receptor complex signalling, and ABA-mediated transcription, with essential roles for the ABSCISIC ACID INSENSITIVE 3 (ABI3) and ABI4 transcription factors. Our data suggest that the ability of mature Arabidopsis embryos to maintain the ABA seed maturation environment is an important first step in establishing competence for auxin-induced cell totipotency. This finding provides further support for the role of ABA in directing processes other than abiotic stress response.

## Teaser

Molecular–genetic analysis of Arabidopsis mutants revealed a novel role for abscisic acid in promoting auxin-induced somatic embryogenesis at three different levels: biosynthesis, signalling, and downstream transcription.

## Introduction

Plant embryogenesis begins at fertilization with the formation of a totipotent zygote that develops into an embryo within the confines of maternal and filial seed tissues. Embryo development proceeds through defined developmental stages that are characteristic for each plant species. In the model plant *Arabidopsis thaliana*, the first phase of embryo development comprises a period of cell proliferation and morphogenesis, where the basic cell types, tissues, and organs are established (Zhao *et al.*, 2017; Tian *et al.*, 2020a). This phase is driven in part by the plant hormone auxin, which acts as a major instructor of cell identity and patterning (Smit and Weijers, 2015; Zhao *et al.*, 2017; Figueiredo and Köhler, 2018). Thereafter, the embryo enters the maturation phase during which cell division is reduced and storage products accumulate that are used to drive embryo growth during germination (Devic and Roscoe, 2016). During the last phase of development, the desiccation and dormancy phase, the water content of the embryo decreases and the embryo enters a quiescent state (Leprince *et al.*, 2017). The maturation and desiccation phases of embryo development are largely controlled by the plant hormone abscisic acid (ABA) (Yan and Chen, 2017) and by a well characterized network of ABA-dependent transcription factors. Among these are the LAFL [for LEAFY COTYLEDON1 (LEC1), ABSCISIC ACID INSENSITIVE 3 (ABI3), FUSCA3 (FUS3), and LEAFY COTYLEDON2 (LEC2)] and ABI4 and ABI5 transcription factors. Mutants of these transcription factors are characterized by a reduction in storage product accumulation and/or desiccation tolerance, but also by the failure to maintain embryo identity (Brocard-Gifford *et al.*, 2003; Carbonero *et al.*, 2016; Devic and Roscoe, 2016; Skubacz *et al.*, 2016; Lepiniec *et al.*, 2018). Seed dormancy can be broken in response to specific environmental signals and by hydration of the seed. During germination, ABA levels decline to promote the transition from embryo development to seedling development (Shu *et al.*, 2016b).

Plant cells are developmentally flexible, and many plant cells other than the zygote can develop into embryos, either naturally as part of an altered seed development programme (León-Martínez and Vielle-Calzada, 2019) or when induced *in vitro* (Soriano *et al.*, 2013; Horstman *et al.*, 2017a; Testillano, 2019). Somatic embryogenesis (SE) is a type of cell totipotency in which embryos develop from vegetative tissues of the plant (Méndez-Hernández *et al.*, 2019; Schmidt, 2020). SE can be induced *in vitro* by exposing explants to exogenous plant growth regulators, usually synthetic herbicidal auxins such as 2,4-dichlorophenoxyacetic acid (2,4-D), often with an additional abiotic stress treatment (Fehér, 2015; Nic-Can *et al.*, 2016). SE forms the basis for a number of plant breeding and biotechnology applications, including clonal propagation (Park *et al.*, 1998; Egertsdotter *et al.*, 2019), but is also used as a model system to understand cell fate changes, in particular in Arabidopsis (Horstman *et al.*, 2017a). SE protocols have been developed for a wide range of Arabidopsis explants, which show different levels of competence and follow different developmental routes to somatic embryo development, including directly from the explant, indirectly through callus, and by secondary SE (Luo and Koop, 1997; Gaj, 2001; Ikeda-Iwai, 2002; Ikeda-Iwai *et al.*, 2003; Wei *et al.*, 2006; Kobayashi *et al.*, 2010; Horstman *et al.*, 2017a).

At present it is not known whether these different routes to SE represent a single pathway or multiple pathways that converge at different downstream points. Nonetheless, a general framework for somatic embryo induction has been proposed in which chromatin-modifying proteins, transcription factors, stress response, and exogenous growth regulator pathways converge at the level of endogenous hormone production and signalling to reprogramme cells to a totipotent state (Fehér, 2015; Horstman *et al.*, 2017a; Pasternak and Dudits, 2019). Direct links between embryo repressive chromatin-modifying proteins and their downstream embryo identity transcription factor genes have been established in Arabidopsis seedlings (Jia *et al.*, 2013; Yang *et al.*, 2013; Chen *et al.*, 2018), as have links between embryo identity transcription factors and endogenous hormone production (Horstman *et al.*, 2017a; Wójcik *et al.*, 2020; Wójcikowska *et al.*, 2020), However, it is not clear how stress modulates SE. With the exception of *Daucus carota* (Kamada *et al.*, 1989, 1993; Nishiwaki *et al.*, 2000), stress treatments on their own are not sufficient to induce SE. Rather, abiotic stress appears to act as an enhancer of plant growth regulator-induced SE (Ikeda-Iwai *et al.*, 2003; Gaj, 2004). In addition to its role as a developmental regulator (Nambara *et al.*, 2010; Hong *et al.*, 2013; Yamaguchi *et al.*, 2018; Yoshida *et al.*, 2019), ABA has key roles as an integrator and modulator of abiotic stress response (Vishwakarma *et al.*, 2017). It has been suggested that an ABA stress response is an important component of competence for SE, as changes in ABA levels and ABA-related gene expression can be associated with competence for SE (Gaj *et al.*, 2006; Su *et al.*, 2013; Fehér, 2015; Kadokura *et al.*, 2018). In Arabidopsis, ABA enhances 2,4-D-induced SE from otherwise non-embryogenic seedling root explants of the POLYCOMB REPRESSIVE COMPLEX 2 CURLY LEAF/SWINGER mutant (*clf swn*) through an unknown mechanism (Mozgová *et al.*, 2017), and modulates auxin response and transport during 2,4-D-induced secondary SE from embryogenic callus (Su *et al.*, 2013). It is not clear whether ABA is required during SE in its role as a developmental regulator or as a stress response modulator. Neither is it known which ABA signalling components have roles during SE.

In this study, we show that 2,4-D-induced SE from the shoot apex of germinating after-ripened Arabidopsis embryos is characterized by the maintenance of an ABA-dependent seed maturation environment. We show genetically that not only ABA, but also ABA perception, signalling, and transcriptional output are required for efficient 2,4-D-induced SE. We also show that the AUXIN RESPONSE FACTORS (ARFs) ARF10 and ARF16, which act upstream and downstream of ABI3 expression, are also required for efficient SE. These data provide a mechanistic link between 2,4-D and ABA signalling in somatic embryo induction, and suggest a developmental role for ABA in promoting plant cell totipotency.

## Materials and methods

### Plant materials and growth

The mutant, reporter, and overexpression lines used in this study are described in Supplementary Table S1. Primers used for cloning and genotyping are listed in Supplementary Table S2.

The *35S:PYL10* vector was made by amplifying the *PYL10* protein-coding sequence from Col-0 genomic DNA and then inserting it into the pGD625 binary vector by Gateway cloning (Immink *et al.*, 2002). The *35S:ABI3* vector was made by amplifying the *ABI3* protein-coding sequence from Col-0 cDNA and then inserting it into the pH7GW2 binary vector (Karimi *et al.*, 2002) using Gateway cloning. The *pBBM:BBM-GFP-GUS* construct was made using a Col-0 PCR fragment containing 4200 bp upstream of the translational start codon up to the end of the *BBM* coding region. This PCR fragment was cloned into the pARC175 binary vector by Gateway cloning (Karimi *et al.*, 2002), which also contains the *GFP-GUS* (green fluorescent protein–β-glucuronidase) reporter and the FAST-Red (*OLEO:OLE01:RFP*) cassette for seed selection (Castel *et al.*, 2019). The FAST-Red cassette was introduced into the pARC175 vector in between the *Xab*I and *Spe*I restriction sites.

*PYL10* CRISPR/Cas9 [clustered regularly interspaced palindromic repeats/CRISPR-associated protein 9] mutagenesis was performed by combining four guide RNAs in the pAGM4723 vector using Golden Gate cloning, as described in Wang *et al.* (2019).

Arabidopsis Col-0 transgenics were obtained by *Agrobacterium tumefaciens*-mediated floral dip transformation (Clough and Bent, 1998), except for the *PYL10* crispants, which were generated by *A. tumefaciens*-mediated root transformation (Vergunst *et al.*, 1998). The *pyl8-1*/*pyl10*^*CR*^ and *pyl8-1*/*pyl9*/*pyl10*^*CR*^ mutants were generated by crossing *pyl8-1* and *pyl8-1*/*pyl9* with *pyl10*^*CR*^, respectively.

All plants were grown in a growth chamber with 70% relative humidity at 20 °C on rock wool cubes (Grodan), which were supplemented twice a week with 1 g l^–1^ 6.5-6-19 liquid fertilizer (Hyponex). The *snrk2.2snrk2.3snrk2.6* triple mutant seedlings and plants were covered with a plastic cap to maintain a high humidity level (Fujii and Zhu, 2009). Plants were maintained under LED light (150 μmol m^–2^ s^–1^) on a 16 h light/8 h dark day/night cycle. Slight differences in plant growth conditions and age at seed harvest can affect the efficiency of somatic embryo cultures (Wu *et al.*, 2019), therefore wild-type control and mutant lines for any given experiment were always grown and harvested at the same time. Unless otherwise indicated, siliques were harvested when they were completely brown and then dried to 30% relative humidity (Wu *et al.*, 2019).

### Somatic embryo culture

Seeds were surface sterilized with liquid bleach and then added to 30 ml of 1/2 MS-10 medium [half-strength Murashige and Skoog macro- and microelements and vitamins (Murashige and Skoog, 1962; Duchefa), 1% (w/v) sucrose, pH 5.8] supplemented with 1 μM 2,4-D (Duchefa) in 190 ml plant tissue culture containers (Greiner). Approximately 60–100 seeds per container were used. The containers were placed at 4 °C in the dark for 2 d and then placed on a shaker (130 rpm) at 25 °C on a 16 h/8 h day/night cycle (100 μmol m^–2^ s^–1^). SE efficiency and productivity were determined after 2 weeks of culture by counting, respectively, the number of seedlings that formed embryogenic tissues or bipolar somatic embryos, and the number of explants with more than two somatic embryos. For some experiments, explants were transferred after 2 weeks of culture to 1/2 MS-10 medium without 2,4-D to promote embryo elongation and thereby facilitate scoring. The results for three technical replicates (same seed batch) are shown for each experiment, and are in agreement with numerous experiments with biological replicates from independent seed batches.

For the ABA treatments, a mixture of ±ABA stereoisomers (Sigma) was dissolved in DMSO and added to the SE culture medium prior to or immediately after stratification or at the indicated time during culture, and then left in the medium for the duration of the culture. The same volume of DMSO was added to control cultures.

### Gene expression analysis

Arabidopsis Col-0 seeds were surface sterilized and grown in containers as described above, with or without 1 μM 2,4-D. The seeds were stratified at 4 °C in the dark for 2 d and then grown for 2 d on a 16 h/8 h day/night cycle at 25 °C on a shaker platform at 130 rpm.

Total RNA was isolated with the Invitrap Plant Spin RNA Mini Kit (Invitek), treated with DNase I (Invitrogen), and sent to the Nottingham Arabidopsis Stock Centre (NASC, http://arabidopsis.info/) for hybridization to the Arabidopsis Affymetrix GeneChip ATH1-121501 microarrays. Three biological replicates were used for the 2,4-D and control treatments.

Raw data were analysed using R Bioconductor packages (www.bioconductor.org; Gentleman *et al.*, 2004). The raw array data were normalized using a robust multichip average (RMA) normalization, which was carried out using the affy package (Gautier *et al.*, 2004). Probe sets that were differentially expressed were identified with linear models generated with limma using a Benjamin and Hochberg adjustment for multiple testing [false discovery rate FDR)] for calculation of the adjusted *P*-values (FDR values) (Ritchie *et al.*, 2015). Gene Ontology (GO) analysis of differentially expressed genes induced by 2,4-D was performed by using DAVID with EASE score (*P*-value <0.05) (Huang *et al.*, 2009).

Quantitative reverse transcription–PCR (qRT–PCR) was performed using RNA isolated with a cetyltrimethylammonium bromide (CTAB)/LiCl protocol and treated with DNase (TURBO DNA-free kit; Invitrogen). cDNA synthesis was performed with the iScript cDNA synthesis kit (BioRad). qRT–PCR was performed as previously described (Horstman *et al.*, 2017b) using the primers shown in Supplementary Table S2. Relative gene expression was calculated according to the 2^−ΔΔ*C*T^ method (Livak and Schmittgen, 2001) using wild-type or DMSO-treated samples as the calibrator (as indicated) and the *SAND* family gene (At2g28390) and the *TIP41*-like gene (At4g34270) (Czechowski *et al.*, 2005) as the reference.

### Histochemistry

GUS activity was determined histochemically as previously described (Soriano *et al.*, 2014), using 1.0–2.5 mM potassium ferri- and ferrocyanide and up to 24 h incubation time. Explants and seedlings were cleared with 70% ethanol prior to imaging.

Neutral lipids were visualized by Sudan Red staining (Sudan Red 7B, Sigma) (Brundrett *et al.*, 1991). Whole explants were incubated for 1 h in filtered Sudan Red solution (0.5% Sudan Red in 60% isopropanol) at room temperature, followed by three washes with water.

Light images were recorded as described below.

### Microscopy

For confocal laser scanning microscopy, seedlings were embedded in 0.2% agarose containing 10 µM FM4-64 (Invitrogen) (de Folter *et al.*, 2007) and imaged with a Leica SPE DM5500 upright confocal microscope using the LAS AF 1.8.2 software. GFP and FM4-64 were excited with a 488 nm and 532 nm solid-state laser, respectively, and emissions were detected at band widths of 500–530 nm and 617–655 nm, respectively.

Light images of explants from SE culture were taken with a Nikon DS-Fi1 camera mounted on a ZEISS Stemi SV 11 binocular. Images were processed with NIS-Elements D 3.2 software.

## Results

### 2,4-D induces SE from the shoot apical meristem (SAM) of germinating seeds

Contrary to a recent report (Wang *et al.*, 2020), embryos from mature, after-ripened Arabidopsis seeds can be readily reprogrammed from seedling development to somatic embryo development by culturing them in 2,4-D (Mordhorst *et al.*, 1998; Thakare *et al.*, 2008; Kobayashi *et al.*, 2010; Wu *et al.*, 2019; Tian *et al.*, 2020a). Here we followed the development of somatic embryos from mature, after-ripened seed explants treated with 1 µM 2,4-D (Wu *et al.*, 2019) by using morphological and embryo identity markers to define the major developmental steps in this process.

**Fig. 1.**
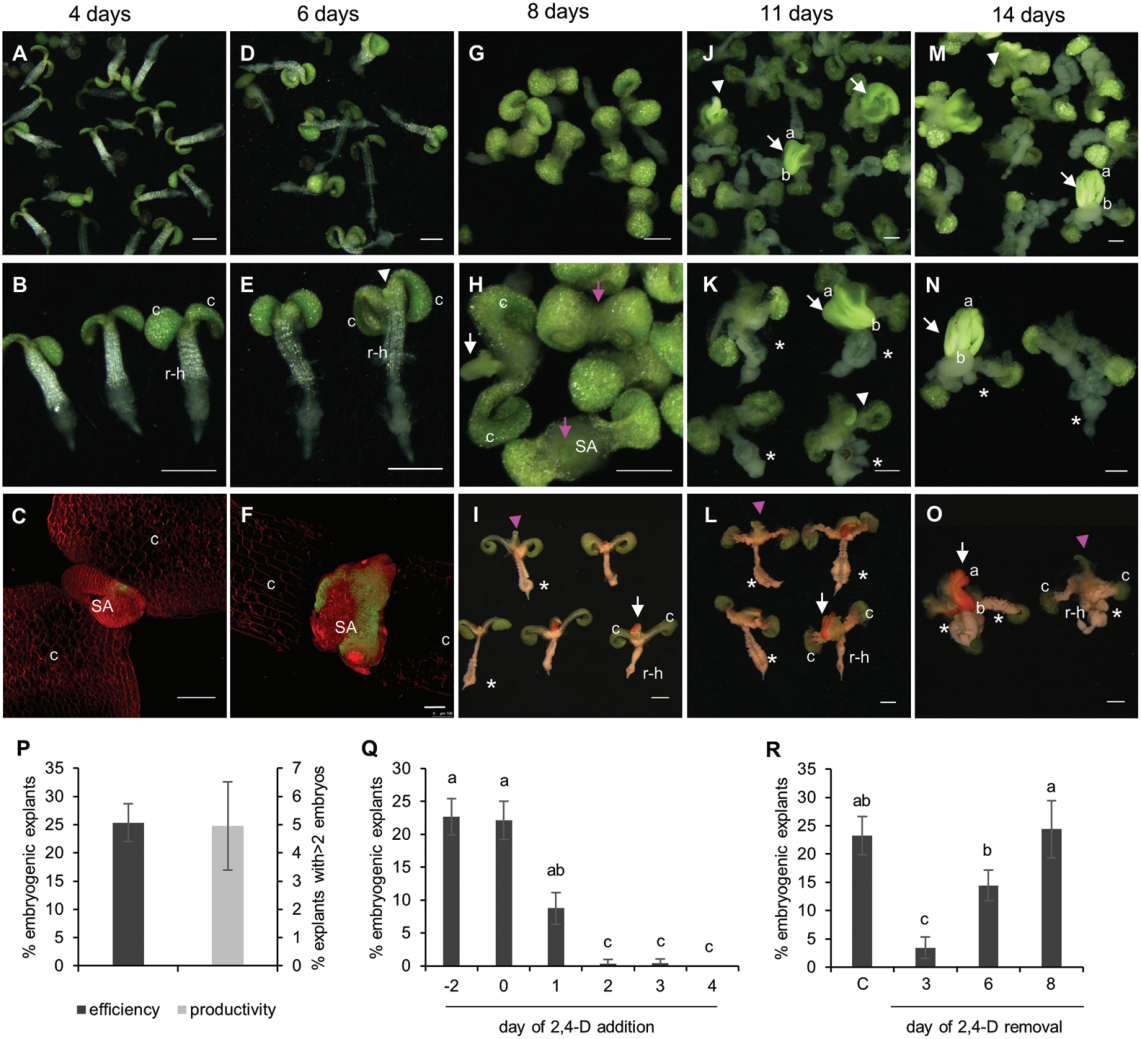
2,4-D-induced somatic embryogenesis (SE) from wild-type mature after-ripened embryo explants. The time of culture is indicated above the panels. (A–N) Overview of somatic embryo cultures in time. (B, E, H, K, N) Magnified images. The images are light micrographs. (C, F) *LEC1:LEC1-GFP* explant showing LEC1–GFP expression (green) at the shoot apex. The explants were counterstained with FM4-64 (red). The images are confocal laser scanning micrographs. (I, L, O) Sudan Red-stained explants. Sudan Red stains the bright green structures and embryos at the shoot apex, but not the ectopic leaf-like structure that develops at the shoot apex of non-embryogenic explants. The images are light micrographs. (A–O) c, cotyledon; r-h, root–hypocotyl; a, apical pole; b, basal pole; white arrowhead, embryogenic structures; white arrow, somatic embryos; pink arrowhead, leaf-like structure; pink arrow, non-embryogenic shoot apex; asterisk, callus. The scale bars are 1 mm in (A), (B), (D), (E), and (G–O), and 100 µm in (C) and (F). (P) Somatic embryogenesis efficiency (percentage of explants with embryogenic tissues and/or bipolar embryos) and productivity (percentage of explants with >2 bipolar embryos) from germinating seeds. (Q) Effect of 2,4-D addition on SE. 2,4-D was added during stratification (–2), at the start of culture (0), or at the indicated time points (1–4) after the start of cultures. (R) Effect of 2,4-D removal on SE induction. 2,4-D was added during stratification (–2) and then removed at the indicated time points by refreshing the medium. C, continuous 2,4-D treatment was used as a control. For (Q) and (R), statistically significant differences in SE efficiency were calculated using Fisher’s least significant difference test. Error bars represent the SD of three technical replicates in one experiment.

During the first 4 d of culture, the seedling cotyledons and petioles enlarged and the epidermal and cortex cells of the root elongation zone and the hypocotyl expanded and began to detach from the underlying tissue (Fig. 1A, B; Supplementary Fig. S1A). Embryogenic and non-embryogenic explants could not be distinguished morphologically on the fourth day of culture, but small patches of *LEC1:LEC1-GFP* embryo reporter expression could already be observed at the enlarged shoot apex of some explants (Fig. 1C). By 6 d of culture, the majority of explants had an elongated hypocotyl–root region, in which the epidermal and cortical cell layers had completely detached from the vascular cylinder above the root meristem (Fig. 1D, E). At this time, cytoplasmic dense, bright green embryogenic protrusions (Fig. 1E) (Verdeil *et al.*, 2007; Godel-Jedrychowska *et al.*, 2020) with LEC1–GFP expression (Fig. 1F) were observed at the shoot apex. The absence of callus at the shoot apex suggests that somatic embryos are formed directly from the shoot apex. Bipolar somatic embryos were visible at the shoot apex from day 8 of culture onward (Fig. 1G, H), but could be most clearly distinguished morphologically during days 11–14 of culture (Fig. 1J, K, M, N; Supplementary Fig. S1C). In addition to expressing LEC1–GFP, these embryogenic protrusions and bipolar somatic embryos were intensely stained by Sudan Red, a dye that stains neutral lipids including the triacylglycerols that accumulate to high levels in Arabidopsis zygotic embryos (Fig. 1I, L, O) (Brundrett *et al.*, 1991). In non-embryogenic explants, the shoot apex either failed to develop or formed a (fused) leaf-like structure (Fig. 1I, J–O). These leaf-like structures were not stained by Sudan Red (Fig. 1I, L, O). Non-embryogenic callus developed in both embryogenic and non-embryogenic explants on the abaxial surface of the cotyledon petiole, under the shoot apex, and from the root–hypocotyl vascular cylinder (Fig. 1G–O; Supplementary Fig. S1B). SE efficiency and productivity were calculated after 14 d of culture (Fig. 1P). SE was induced in ~25% of the explants (SE efficiency) of which ~5% developed more than two bipolar embryos (SE productivity).

We determined the developmental window in which 2,4-D is required to induce SE in germinating embryos by adding or removing 2,4-D at different time points in culture (Fig. 1Q, R). Addition of 2,4-D during seed stratification at 4 °C or at the start of culture induced the highest SE efficiency, while adding 2,4-D at progressively later time points decreased SE efficiency, such that SE could no longer be induced when 2,4-D was added after the third day of culture (Fig. 1Q). Removal of 2,4-D after 3 d of culture dramatically decreased somatic embryo induction, while removal at later time points only had a mild effect on SE efficiency compared with continuous treatment.

These results indicate that treatment of mature after-ripened embryos with 2,4-D inhibits normal shoot apex development to promote embryogenesis, and that the developmental competence for shoot apex embryogenesis is established within 48 h of culture. These results are in contrast to previous reports showing loss of SE competence in mature embryo explants within 1 d after germination (Mozgová *et al.*, 2017), but might reflect differences in the type of SE under study (direct versus indirect).

### 2,4-D maintains the seed ABA maturation pathway post-germination

**Fig. 2.**
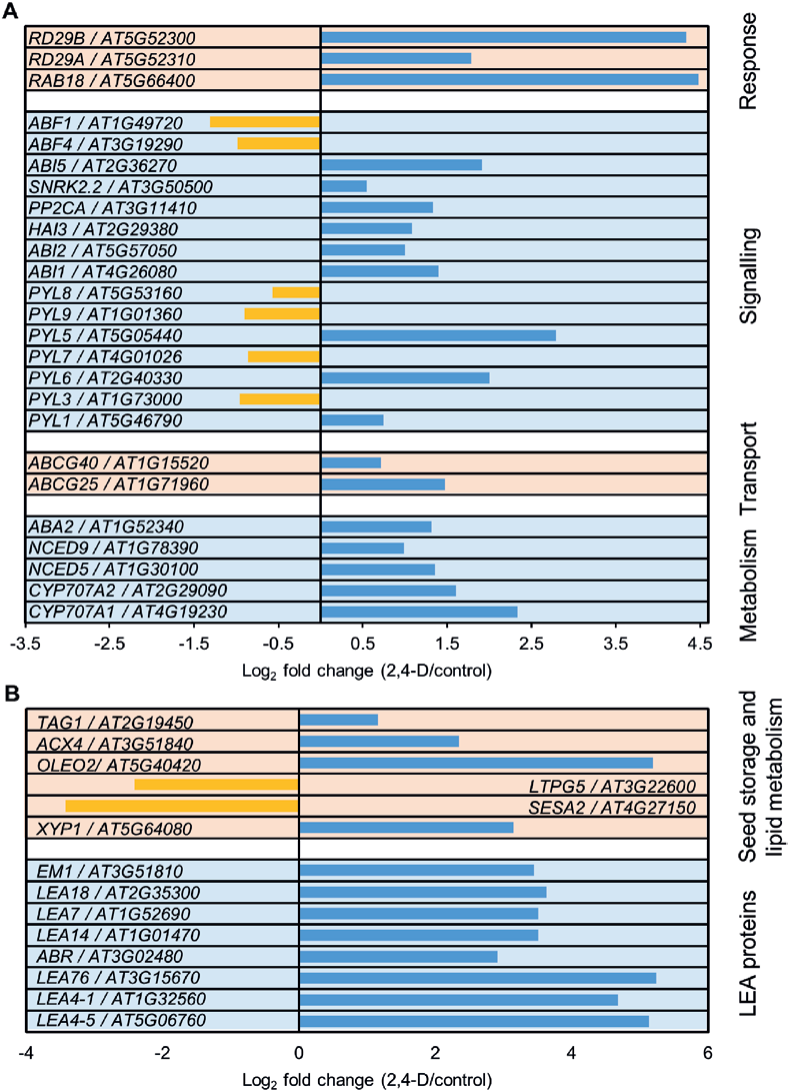
2,4-D promotes ABA-related gene expression post-germination. (A) Selection of statistically significant differentially expressed ABA-related genes. (B) Selection of statistically significant differentially expressed seed maturation genes. In (A) and (B), the gene name and Arabidopsis gene identifier (AGI), as well as the log_2_ fold expression change for 2,4-D-treated versus control seedlings are shown for each gene. Genes were grouped per functional category. The complete dataset can be found in Supplementary Data Set S1.

To identify the signalling pathways that are affected by 2,4-D treatment, we compared the transcriptomes of imbibed seeds cultured for 48 h in medium with or without 1 µM 2,4-D. We identified 5687 and 5300 genes that were significantly up- or down-regulated, respectively, by 2,4-D compared with the untreated control (log_2_ fold change >0.5 or < –0.5, FDR <0.05; see Supplementary Data Set S1). GO analysis of both up- and down-regulated genes revealed that changes in the expression of genes involved in response to cadmium ion, salt stress, cytokinin, auxin signalling, homeostasis, and response were among the most highly enriched categories (Supplementary Figs S2, S3; Supplementary Data Set S1). The 2,4-D treatment also induced statistically significant changes in expression of genes involved in ABA, dehydration, and cold stress, and seed maturation pathways (Fig. 2A, B; Supplementary Fig. S2; Supplementary Data Set S1). The expression of these seed-expressed ABA and maturation-related genes is normally down-regulated during the transition to germination (Carles *et al.*, 2002; Lopez-Molina *et al.*, 2002; Cadman *et al.*, 2006; Nakashima *et al.*, 2006; Braybrook and Harada, 2008; Yamaguchi *et al.*, 2018), suggesting that 2,4-D treatment maintains the ABA seed maturation pathway post-germination. The differential expression of selected auxin and ABA pathway genes was confirmed by qRT–PCR analysis (Supplementary Fig. S3, Supplementary Fig. S4).

A number of Arabidopsis genes have been identified that induce spontaneous SE when ectopically expressed and/or enhance 2,4-D-induced SE (Horstman *et al.*, 2017a). We therefore examined whether any of these genes are differentially expressed within the first few days of somatic embryo induction (Supplementary Data Set S1). Surprisingly, of these genes, only *PLT1*, *PLT2*, and *BBM* expression was significantly up-regulated in 2-day-old 2,4-D-treated explants. However, *BBM:BBM-GUS* reporter analysis (Supplementary Fig. S5) showed that *BBM* was not expressed at the shoot apex in 2-day-old explants, but was restricted to its normal expression domain in the root meristem (Galinha *et al.*, 2007). *BBM:BBM-GUS* activity was only observed in the shoot apex of embryogenic explants from 6 d of culture onward. These data suggest that expression of somatic embryo identity genes is enhanced by 2,4-D in their natural expression domain in the root, followed later by ectopic expression in the shoot meristem. The relatively late expression of those embryo identity genes in the shoot meristem suggests that other developmental changes precede expression of SE-inducing transcription factors in the shoot meristem.

### Endogenous ABA is required for efficient somatic embryo induction

**Fig. 3.**
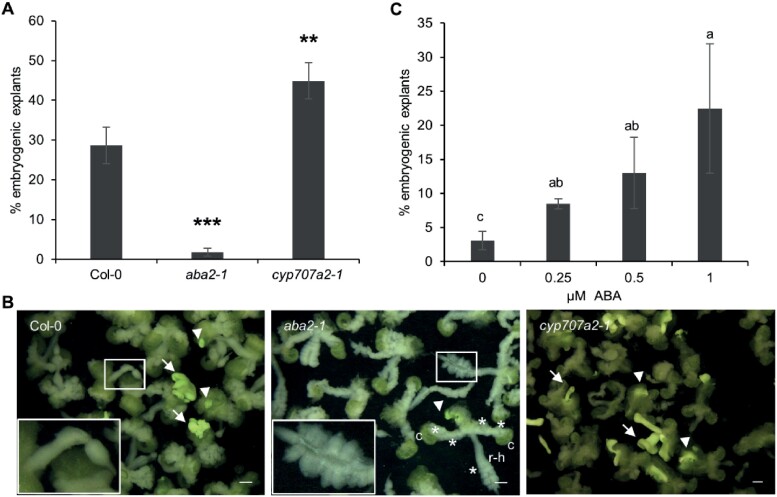
Endogenous ABA is required for efficient somatic embryogenesis. (A) The effect of ABA biosynthesis mutants on SE. Statistically significant differences in SE efficiency between the wild type and the mutant lines were calculated using a two-tailed Student’s *t*-test (**P<0.01; ***P<0.001). Error bars represent the SD of three technical replicates. (B) Light images of 14-day old explants from somatic embryo cultures of the indicated wild-type and mutant lines. The insets in the wild-type and *aba2-1* panels are magnifications of the respective boxed regions, showing excessive callus formation at the root–hypocotyl region of the explant of the *aba2-1* mutant. The images are light micrographs. Arrows, somatic embryos; arrowheads, embryogenic tissue; asterisks, callus; c, cotyledon; r-h, root–hypocotyl. Scale bars, 1 mm. (C) Application of exogenous ABA restores SE efficiency to wild-type Col-0 levels in the *aba2-1* mutant. The Col-0 control was previously reported by Wu *et al.* (2019). Statistically significant differences in SE efficiency were calculated using Fisher’s least significant difference test. Error bars represent the SD of three technical replicates.

2,4-D-treated somatic embryo cultures showed up-regulation of ABA pathway genes that are normally expressed during embryo maturation and down-regulated during embryo germination (Fig. 2). ABA/ABA stress signalling has been proposed to promote SE, but the mechanism has not been well characterized. We therefore focused our subsequent analysis on the role of ABA in SE from the shoot apex of germinating embryos. Previously we showed that ABA biosynthesis is important for SE competence in fresh mature seeds (harvested from yellow siliques that are dried at 30% relative humidity and then stored at –80 °C) and aged seeds (stored at room temperature for 5 years) (Wu *et al.*, 2019). The ABA biosynthesis mutant, *aba2-1*, which has reduced endogenous ABA levels (Leon-Kloosterziel *et al.*, 1996; González-Guzmán *et al.*, 2002), negatively affected SE efficiency in both fresh mature and aged seeds, while the *cyp707a2-1* mutant, which has a higher endogenous ABA level (Kushiro *et al.*, 2004), enhances SE efficiency (Wu *et al.*, 2019). We obtained similar results with these mutants using mature after-ripened seed explants (Fig. 3). Compared with wild-type explants, the *cyp707a2-1* mutant enhanced SE efficiency and the *aba2-1* mutant reduced SE efficiency (Fig. 3A). The *aba2-1* mutant also developed more callus on the cotyledon petioles, under the shoot apex, and throughout the root–hypocotyl region than wild-type explants (Fig. 3B). The reduction in SE efficiency in the *aba2-1* background could be fully complemented by addition of 1 µM ABA to the culture medium (Fig. 3C). The mutant phenotypes and ABA complementation experiments indicate that endogenous ABA is required and limiting for efficient SE. However, we have shown previously that treatment of mature after-ripened seed explants with exogenous ABA slightly inhibits SE (Wu *et al.*, 2019). Thus, although ABA levels are limiting for SE from the shoot apex, they also need to be tightly regulated to promote SE.

### The ABA receptor complex positively regulates auxin-induced SE

Given the requirement of endogenous ABA for somatic embryo development from germinating seeds (Fig. 3; Wu *et al.*, 2019), we focused our efforts on identifying the specific components of the ABA signalling pathway (Fig. 6) that are required for this developmental process. ABA is perceived and transduced by a ternary ABA signalling complex comprising RCAR/PYR1/PYL ABA receptors (hereafter referred to as PYLs), clade A PP2C protein phosphatases, and SnRK2 kinases (Fujii *et al.*, 2009; Ma *et al.*, 2009; Park *et al.*, 2009). ABA-bound PYLs interact with and inhibit PP2Cs, which in turn promotes activation of SnRK2 kinases such as SnRK2.2/3/6. The activated SnRK2 protein kinases phosphorylate and activate various downstream substrates, including ABA-responsive transcription factors (Fujii *et al.*, 2009; Melcher *et al.*, 2009; Yin *et al.*, 2009; Raghavendra *et al.*, 2010; Soon *et al.*, 2012; Xie *et al.*, 2012).

**Fig. 4.**
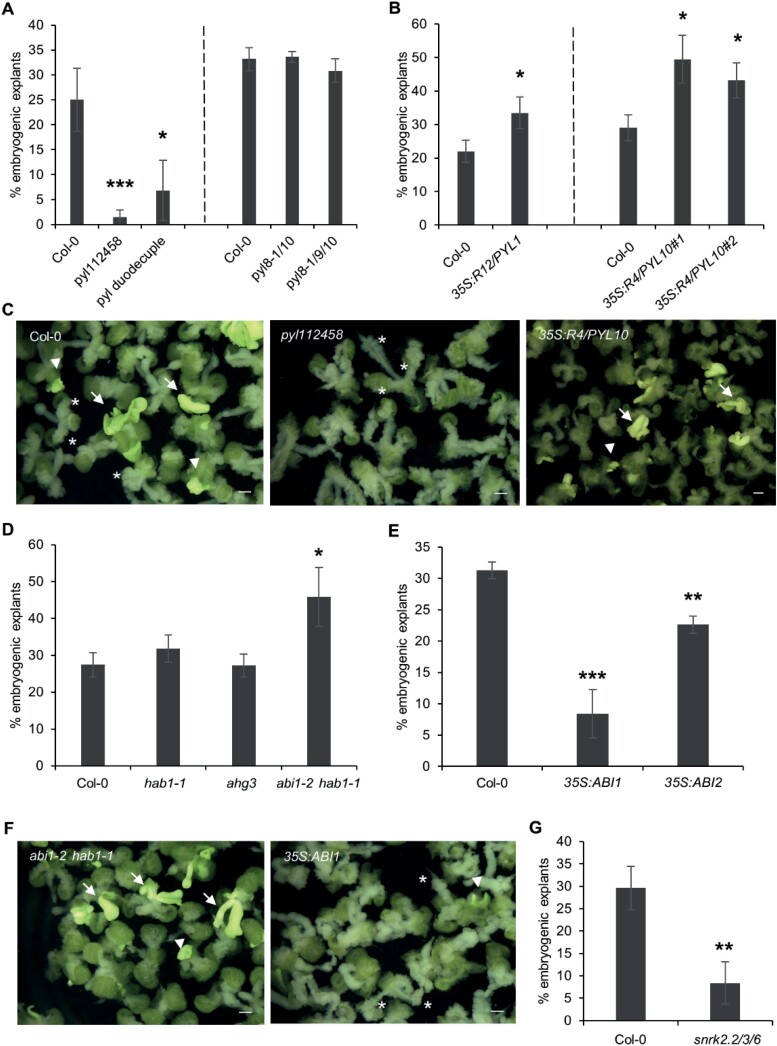
Signalling through the ABA receptor complex positively regulates 2,4-D-induced somatic embryogenesis. (A) Somatic embryogenesis (SE) efficiency in ABA receptor mutants. The corresponding wild-type Col-0 control is shown for each set of mutants. (B) Effect of overexpression of the *RCAR12/PYL1* and *RCAR4*/*PYL10* ABA receptor genes on SE efficiency. Independent experiments are separated by a dashed line in (A) and (B). (C) Overview of 14-day-old wild-type Col-0 and ABA receptor mutant phenotypes in somatic embryo culture. The images are light micrographs. (D) Effect of *PP2C* single and double mutants on SE efficiency. (E) Effect of overexpression of the *PP2C* genes *ABI1* and *ABI2* on SE efficiency. (F) Overview of 14-day-old PP2C mutant phenotypes in somatic embryo culture. The images are light micrographs. (G) Effect of *SnRK2* mutants on SE efficiency. Statistically significant differences in SE efficiency between wild-type Col-0 and mutant explants were calculated using a two-tailed Student’s *t*-test (*P<0.05; **P<0.01; ***P<0.001). Error bars represent the SD of three technical replicates. Arrows, somatic embryos; arrowhead, embryogenic tissue; asterisks, callus. Scale bars, 1 mm.

We first determined whether ABA receptors play a role in somatic embryo induction from germinating seeds. Our transcriptome analysis showed that of the 14 Arabidopsis *PYL* genes, seven were (differentially) expressed in 2,4-D-treated cultures, including *PYL1* (up-regulated) and *PYL3* (down-regulated) in subfamily III, *PYL5* and *PYL6* (both up-regulated) in subfamily II, and *PYL7*, *PYL8*, and *PYL9* (all down-regulated) in subfamily I (Fig. 2A; Supplementary Data Set S1). *PYL10*, *PYL11*, *PYL12*, and *PYL13* are not represented on the ATH1 microarray. Given the functional redundancy between ABA receptors (Park *et al.*, 2009; Zhao *et al.*, 2018), we analysed higher order *RCAR/PYR1/PYL* mutants for their effect on SE. ABA signalling is blocked to a large extent in the *pyl112458* sextuple mutant, which carries T-DNA insertions in the *PYL1*, *PYL2*, *PYL4*, *PYL5*, and *PYL8* genes and a point mutation in *PYR1* (Gonzalez-Guzman *et al.*, 2012). This sextuple mutant had a strong negative effect on somatic embryo formation (Fig. 4A, C) that could not be rescued by exogenous ABA application (Supplementary Fig. S6), suggesting that the requirement for ABA for efficient SE depends on a functional ABA receptor complex. Similarly, the *pyl* duodecuple mutant (*pyl112458379101112*), in which only one functional ABA receptor, *PYL6*, is a wild-type allele (Zhao *et al.*, 2018), also had a negative effect on somatic embryo development (Fig. 4A). The phenotype of these higher order ABA receptor mutants resembled that of the *aba2-1* mutant explants, in which SE efficiency was compromised and non-embryogenic callus formation was stimulated. Of the genes in the *PYL7*, *PYL8*, *PYL9*, *PYL10* subfamily (subfamily I), loss-of-function mutants are only available for *PYL8* and *PYL9*. We therefore made a *PYL10* null mutant using CRISPR/Cas9 mutagenesis (*pyl10*^*CR*^) and crossed this mutant with *PYL8* and *PYL9* null mutants to obtain double and triple mutants (*pyl8-1pyl10*^*CR*^ and *pyl8-1pyl9pyl10*^*CR*^). The *pyl8-1pyl10*^*CR*^ and *pyl8-1pyl9pyl10*^*CR*^ mutants did not show obvious mutant phenotypes during somatic embryo culture (Fig. 4A). In contrast to the negative effect of the loss-of-function *pyl* mutants on SE, overexpression of both *RCAR12/PYL1* (*35S:RCAR12/PYL1*; Yang *et al.*, 2016) and *RCAR4/PYL10* (*35S:RCAR4/PYL10*) (Fig. 4B, C; Supplementary Fig. S7) slightly, but significantly, enhanced SE efficiency. Together, the data from both loss of function and overexpression of ABA receptor complex components suggest that functional ABA receptors are required for efficient SE and that enhanced basal ABA signalling promotes 2,4-D-induced SE.

Next, we determined whether mutants for PP2C protein phosphatases (negative ABA signalling regulators) and SnRK2 protein kinases (positive ABA signalling regulators) affect 2,4-D-induced somatic embryogenesis from germinating seeds. *ABI1*, *ABI2*, and *AHG3/PP2CA* are up-regulated in our 2,4-D-treated seed explants (Fig. 2A; Supplementary Data Set S1). These genes are known to play roles in ABA-mediated repression of seed germination together with the PP2C phosphatase gene *HAB1* (Rubio *et al.*, 2009). SE efficiency was not affected in the *hab1-1* or *ahg3* single mutants, while the higher order *abi1-2 hab1-1* PP2C mutant showed enhanced SE efficiency (Fig. 4D, F). Accordingly, overexpression of either *ABI1* (*35S:ABI1*) or *ABI2* (*35S:ABI2*) (Wang *et al.*, 2018) inhibited somatic embryo formation (Fig. 4E, F). Compared with wild-type explants, explants from the *abi1-2 hab1-1* loss-of-function mutant did not show any abnormal phenotypes, while *ABI1* overexpression explants produced more non-embryogenic callus over the entire explant (Fig. 4F).

Among the subclass III SnRK2 kinases, only *SnRK2.2* was up-regulated by 2,4-D treatment (Fig. 2A; Supplementary Data Set S1). *SnRK2.2*, *SnRK2.3*, and *SnRK 2.6* function redundantly in the regulation of ABA-mediated seed germination, therefore the *snrk2.2snrk2.3snrk2.6* triple mutant (Fujii and Zhu, 2009) was evaluated for its effect on SE. This mutant had a strong negative effect on SE from germinating seeds (Fig. 4G). As with other positive regulators of ABA signalling, *snrk* mutant explants produced more non-embryogenic callus than wild-type explants (data not shown).

Together, these data indicate that signalling through the ABA receptor complex, from ABA perception to protein kinase function, is required for 2,4-D-mediated SE from germinating seeds.

### Seed maturation transcription factors positively regulate auxin-induced SE

The *ABI3*, *ABI4*, and *ABI5* transcription factor genes act downstream of ABA signalling and encode, respectively, B3-, APETALA2- (AP2), and basic leucine zipper- (bZIP) domain DNA-binding proteins. These *ABI* genes were identified based on genetic screens for mutants that are insensitive to ABA during seed germination, but were later shown to also have overlapping roles in seed maturation (Parcy *et al.*, 1994; Carles *et al.*, 2002; Penfield *et al.*, 2006; Delmas *et al.*, 2013), in abiotic stress responses during seed germination and seedling growth (Söderman *et al.*, 2000; Lopez-Molina *et al.*, 2001, 2002; Brocard *et al.*, 2002), as well as other post-germination functions (Brady *et al.*, 2003; Shkolnik-Inbar and Bar-Zvi, 2010; Wang *et al.*, 2013). *ABI3*, *ABI4*, and *ABI5* are subject to extensive transcriptional cross-regulation during seed maturation and germination (Söderman *et al.*, 2000; Yan and Chen, 2017). A number of ABI3, ABI4, and ABI5 target genes were up-regulated in the transcriptome dataset, including the LEA genes *LEA4-1*, *LEA76*, *AT5G44310*, and *AT4G21020*, and *PGIP1* and *NYC1* (Fig. 2B; Supplementary Fig. S4; Supplementary Data Set S1; Reeves *et al.*, 2011; Mönke *et al.*, 2012; Skubacz *et al.*, 2016) Given the expression patterns of ABA- and ABI*-*regulated seed maturation pathway genes, we examined the expression and function of the three *ABI* genes during SE. Microarray-based transcriptome analysis showed that of these three genes, only *ABI5* expression was up-regulated in 2,4-D-treated mature after-ripened seeds (Fig. 2A; Supplementary Data Set S1), but qRT–PCR analysis showed that *ABI3* and *ABI4* expression was also up-regulated significantly at this time point (Supplementary Fig. S4).

**Fig. 5.**
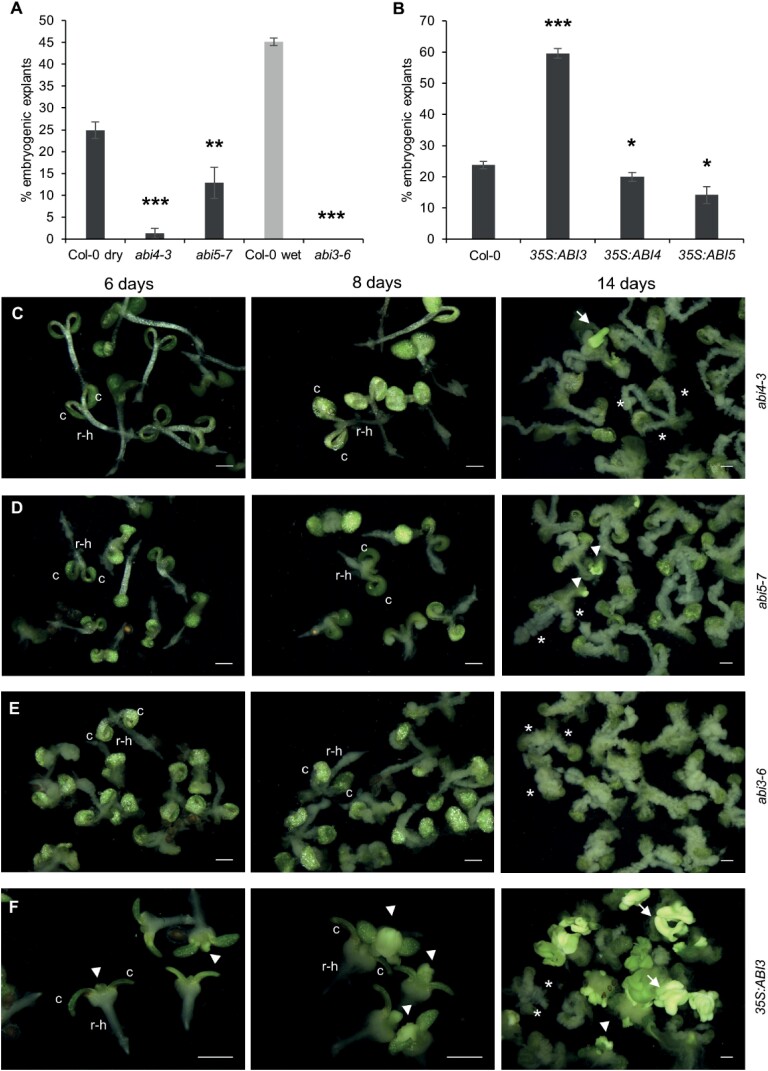
Seed maturation transcription factors are required for efficient somatic embryogenesis (SE). (A) Effect of *ABI4*, *ABI5*, and *ABI3* loss-of-function mutants on 2,4-D-induced SE. The *abi3-6* mutant is desiccation intolerant, therefore mature wet seeds were analysed for this mutant, using mature wet Col-0 seeds as a control. (B) Effect of *ABI3*, *ABI4,* and *ABI5* overexpression on 2,4-D-induced SE. (C–F) Overview of somatic embryo cultures in time for the indicated mutants. The images are light micrographs. The day of culture is indicated above the panels. Statistically significant differences in SE efficiency between wild-type Col-0 and mutant explants were calculated using a two-tailed Student’s *t*-test (*P<0.05; **P<0.01; ***P<0.001). Error bars represent the SD of three technical replicates. c, cotyledon; r-h, root–hypocotyl region; arrow, somatic embryos; arrowheads, embryogenic tissues; asterisks, callus formed on the petioles or the root–hypocotyl region. Scale bars, 1 mm.

Next, we examined the effect of *ABI3*, *ABI4*, and *ABI5* mutant and overexpression lines on 2,4-D-induced somatic embryo induction. *abi3* null mutants are desiccation intolerant; therefore, we analysed the response of weak *abi3* alleles (*abi3-8*, *abi3-9*, and *abi3-10*; Nambara *et al.*, 2002) using mature, after-ripened seed explants, and the response of the *abi3-6* null allele (Nambara *et al.*, 1994) using mature wet seed explants. All *abi* mutants displayed a strong reduction in SE efficiency from mature seed explants, with the *abi3-6* (0%) and *abi4* mutants (2–5%) being most severely affected (Fig. 5A–E; Supplementary Fig. S8). Consistent with our results on the core ABA signalling complex, *abi3*, *abi4*, and *abi5* explants also produced more non-embryogenic callus than wild-type explants (Fig. 5C–E). The higher SE response in the *abi3-8*, *abi3-9*, and *abi3-10* mutants compared with the *abi3-*6 mutant might be due to, respectively, partial versus complete loss of function of the mutant alleles (Nambara *et al.*, 2002; Delmas *et al.*, 2013). The higher SE responses in *abi5-7* compared with *abi4-3* and *abi3-6* might be due to functional redundancy of ABI5 with another eight ABRE-binding factors (ABFs). The number of embryogenic explants was also severely reduced in the weak *abi3* mutants and in the *abi4* and *abi5-7* mutants.

In wild-type immature zygotic embryo explants, prolific SE takes place on the adaxial surface of the cotyledon petiole, while a single somatic embryo develops from the shoot apex (Supplementary Fig. S9A). *abi3-6* immature zygotic embryo explants produced somatic embryos on the adaxial surface of the petiole, as in wild-type explants, but, unlike wild-type explants, a shoot-like structure developed from the apex instead of a somatic embryo (Supplementary Fig. S9B). These results indicate that *abi3-6* immature zygotic embryo explants retain the ability to form somatic embryos from the petiole, but not from the shoot apex (Supplementary Fig. S9C), and suggest that ABI3 is required to repress shoot development from the shoot apex.

We determined whether exogenous ABA application could improve SE efficiency in the *abi3*, *abi4*, and *abi5* mutant backgrounds. ABA application did not improve SE efficiency in the *abi3-6*, *abi4-1*, or *abi4-2* backgrounds, but did have a positive effect on SE in the *abi5-7* background (Supplementary Fig. S10), suggesting that auxin-induced SE is less dependent on *ABI5* than on *ABI3* and *ABI4*.

Next, we examined the effect of *ABI3*, *ABI4*, and *ABI5* ectopic overexpression on somatic embryo formation using *35S:ABI3* (Supplementary Fig. S7), *35S:ABI4*, and *35S:ABI5* lines (Brocard *et al.*, 2002; Shu *et al.*, 2013). *ABI3* overexpression enhanced, while *ABI4* and *ABI5* overexpression reduced, SE efficiency in 2,4-D-treated cultures (Fig. 5B, F). 2,4-D-treated *35S:ABI3* explants had a larger embryogenic shoot apex than 2,4-D-treated wild-type explants, which was already visible after 4 d of culture compared with 6 d in wild-type explants (compare Fig. 1E and Fig. 5F). A similarly enlarged shoot apex was not observed in 2,4-D-treated *ABI4* and *ABI5* overexpression lines.

Collectively, our genetic data showed that ABI3, ABI4, and ABI5 positively regulate auxin-induced SE from germinating seeds. SE efficiency was reduced in the *abi5-7* mutant, even more so in the *abi4-3* mutant, and was completely abolished in the *abi3-6* mutant. The complete absence of embryogenic growth in the *abi3-6* mutant and the positive effect of *ABI3* overexpression on SE suggests that *ABI3* expression is essential and limiting for somatic embryo initiation. The slight negative effect of *ABI4* and *ABI5* overexpression on SE efficiency might reflect additional stress signalling roles for these proteins that interfere with SE. Exogenous ABA application enhanced SE in the *abi5* loss-of-function mutant, but not in the *abi3* and *abi4* loss-of-function mutants. Together, these data suggest that *ABI3* and *ABI4* are the main downstream effectors of ABA signalling during SE.

ABI3 is a key component of 2,4-D-induced SE from mature after-ripened embryos. Auxin signals through the ARFs ARF10 and ARF16 to maintain *ABI3* expression and enhance ABA-mediated inhibition of seed germination (Liu *et al.*, 2013). ABI3 also regulates *ARF10* and *ARF16* expression through *MIR160*, which post-transcriptionally regulates *ARF10* and *ARF16* levels (Tian *et al.*, 2020b). Therefore, we determined whether mutations in ARF10 and ARF16 influence 2,4-D-induced SE. The efficiency of 2,4-D-induced SE was significantly reduced in the *arf10/16* double mutant (Supplementary Fig. S12), suggesting that 2,4-D and ABA converge at the level of ARF10 and/or ARF16 and ABI3.

### Developmental timing of *ABI3* and *ABI4* expression in SE culture

ABI3 and ABI4 are strong positive regulators of auxin-induced SE. We examined their expression patterns during the course of somatic embryo induction to better understand their roles in this process. *ABI3* and *ABI4* expression was followed during SE culture using *ABI3:GUS* (Ryu *et al.*, 2014) and *ABI4:GUS* (Söderman *et al.*, 2000) reporter lines (Supplementary Fig. S11). Seeds were cultured with or without 2,4-D, and explants stained for GUS activity over an 8 d period. *ABI3* (Supplementary Fig. S11A) was expressed initially in the cotyledons and the hypocotyl of germinating seeds from both auxin-treated and control seedlings, with higher expression in 2,4-D-treated samples at day 2 of culture. Four days after the start of culture, *ABI3* expression could no longer be detected in control seedlings, but was still present in auxin-treated explants. Later, from day 6 to day 8 of culture, *ABI3:GUS* expression became restricted to the cotyledons and shoot apex (Supplementary Fig. S11A). *ABI4* expression (Supplementary Fig. S11B) was also gradually lost in control seedlings compared with 2,4-D-treated seedlings, although *ABI4* expression declined earlier than *ABI3* expression. No obvious difference in *ABI3*- or *ABI4*-driven GUS activity was found between embryogenic and non-embryogenic explants from 2,4-D-induced somatic embryo culture (Supplementary Fig. S11).

*ABI3* and *ABI4* are induced transiently during seed germination (https://www.bioinformatics.nl/dormancy/). Our reporter, microarray, and qPCR data therefore suggest that *ABI* gene expression is maintained post-germination in response to auxin treatment. The developmental window in which *ABI* expression normally decreases in control seedlings corresponds to the window for efficient 2,4-D-induced SE. *ABI3* and *ABI4* are essential for SE, but GUS reporter analysis suggests that they are not differentially regulated at the transcriptional level between embryogenic and non-embryogenic explants. The lack of difference in expression of these two genes between embryogenic and non-embryogenic explants suggests that *ABI3* and *ABI4* expression is regulated post-transcriptionally in these explants (Zhang *et al.*, 2005; Finkelstein *et al.*, 2011; Gregorio *et al.*, 2014).

## Discussion

The vast majority of SE protocols use 2,4-D as the inducer treatment, alone or in combination with an abiotic stress treatment (Gaj, 2004). 2,4-D treatment has also been shown to induce a transcriptional stress response during SE (Rai *et al.*, 2011; Nic-Can and Loyola-Vargas, 2016; Kadokura *et al.*, 2018). Somatic embryo induction by stress treatment alone has rarely been described (Kamada *et al.*, 1989, 1993; Nishiwaki *et al.*, 2000), suggesting that a stress response is in itself not sufficient for somatic embryo initiation, but rather is needed to enhance the effect of auxin treatment. The role of ABA as a core regulator of diverse plant abiotic stress responses has been well documented in many plant species (Vishwakarma *et al.*, 2017; Cho *et al.*, 2018). It is clear that auxin interacts with the ABA pathway during somatic embryo induction, but it is not clear whether these interactions are stress related or simply reflect developmental roles for ABA in basal signalling pathways (Yoshida *et al.*, 2019). The observation that ABA modulates auxin response and transport during 2,4-D-induced secondary SE from embryogenic callus in Arabidopsis supports a developmental role for ABA during SE (Su *et al.*, 2013), but it is not known which ABA signalling components regulate this response.

Here we show that 2,4-D-induced SE from mature after-ripened Arabidopsis embryos induces a transcriptional cascade that is characteristic for the ABA seed maturation pathway (Fig. 2). Genes in this pathway are normally down-regulated in mature after-ripened seeds or during germination, but their expression is maintained when imbibed seeds are cultured in 2,4-D. We show that ABA promotes and is limiting for SE at three different levels: ABA biosynthesis, ABA receptor complex signalling, and ABA-mediated transcription, and that ABI3 and ABI4 are essential players in this process (Figs 3–5). Our results suggest a novel developmental role for a basal ABA signalling pathway in modulating auxin-dependent cell fate changes in the shoot apex.

### SE requires and is limited by upstream components of the ABA signalling pathway

Endogenous ABA is required for 2,4-D-induced SE from the shoot apex of germinating seeds (Fig. 3). Higher order ABA receptor mutants, where ABA signalling is blocked to a large extent (Gonzalez-Guzman *et al.*, 2012), also show reduced SE efficiency that cannot be complemented by exogenous ABA (Figs 4, 5). Together, these data suggest that a basal ABA level is required for SE and that ABA signals through the RCAR/PYR1/PYL receptor complex to regulate 2,4-D induced SE.

Endogenous ABA is required for 2,4-D-induced (indirect) secondary SE from callus, where it is thought to modulate auxin response and transport during secondary embryo outgrowth (Su *et al.*, 2013). In this system, immature zygotic embryos are cultured in 2,4-D to induce somatic embryo formation and then callus formation, followed by secondary SE from callus after removal of auxin from the medium. Here SE is induced by 2,4-D treatment directly from the SAM of germinating mature after-ripened embryos. Whether ABA-regulated auxin response and transport is a common component of/is required for SE systems that rely on different explants and follow different developmental pathways (primary/secondary, direct/indirect) remains to be determined by genetic analysis.

In line with the transcriptome data showing 2,4-D-induced *PYL1* and *PYL5* up-regulation (Fig. 2A), the higher order loss-of-function mutant (*pyl112458*) in subfamilies I, II, and III negatively affected SE progression (Fig. 4A), while overexpression of PYL1/RCAR12 (subfamily III) enhanced SE (Fig. 4B). Expression of the subfamily I receptor genes *PYL7*, *PYL8*, and *PYL9* was down-regulated by 2,4-D treatment (Fig. 2A), but *pyl8-1*, *pyl9*, and *pyl10*^*CR*^ mutant combinations had no effect on SE efficiency (Fig. 4A). However, we cannot rule out a (different) role for subfamily I RCAR/PYR1/PYL receptors, as genetic redundancy between *PYL7* and the other subfamily I members and/or other receptor subfamilies might mask a role for these genes during SE (Park *et al.*, 2009; Zhao *et al.*, 2018). Although subfamily I RCAR/PYR1/PYL receptors do not appear to have a major role in SE, overexpression of one subfamily 1 receptor, PYL10, did enhance SE efficiency. The ability of *35S:PYL10* to enhance SE might therefore indicate a lack of specificity of the ABA receptors with respect to the downstream signalling pathways that are regulated during 2,4-D-induced SE.

Overexpression of the *ABI1* and *ABI2 PP2C* protein phosphatase genes inhibited SE (Fig. 4E), while the *abi1 hab1* double mutant showed enhanced SE, in line with their role as negative regulators of ABA signalling (Fig. 4D). According to the transcriptome data, expression of the *ABI1*, *ABI2*, and *AHG3/PP2CA PP2C* protein phosphatase genes, which are negative regulators of ABA signalling, was slightly up-regulated by 2,4-D treatment (Fig. 2A). This suggests that up-regulation of these *PP2C* genes after 2,4-D treatment is due to negative feedback regulation that keeps downstream ABA signalling in check (Maia *et al.*, 2014).

Overall, we show dependence on various mediators of ABA biosynthesis and signalling to enhance 2,4-D-induced SE. However, the transcriptome data imply that the ABA transcriptional response is far more complex. For example, genes for both ABA biosynthesis (ABA2, NCED5, and NCED6) and inactivating enzymes (CYP707A1 and CYP707A2) are up-regulated after 2,4-D treatment (Fig. 2A), yet our mutant analysis showed that increased SE potential was correlated with loss of CYP707A2 activity (Fig. 3A). Our transcriptome data were obtained from whole embryos, while only a subset of the explant cells contribute either cell autonomously or non-autonomously to somatic embryo competence. In addition, the time point at which specific genes function during the course of culture needs to be taken into consideration, and can be difficult to define with mutants. Additional studies using RNAi and reporter lines for specific genes, as well as pharmacological intervention (Park *et al.*, 2009; Nemoto *et al.*, 2018), will help to resolve the contributions of the different signalling components to 2,4-D-induced SE.

### Auxin maintains the seed maturation environment

In Arabidopsis, *ABI4* and *ABI5* together with *LAFL* genes (*LEC1*, *ABI3*, *FUS3*, and *LEC2*) control the maturation and desiccation phases of zygotic embryo development (Brocard-Gifford *et al.*, 2003; Carbonero *et al.*, 2016; Skubacz *et al.*, 2016; Lepiniec *et al.*, 2018). Mutant analysis has shown that *LAFL* genes also regulate other aspects of embryo development, including repression of seedling-expressed genes in the early embryo (Yamamoto *et al.*, 2014) and promotion of suspensor and cotyledon development. In line with these functions, ectopic overexpression of these genes in seedlings confers embryo identity traits, and in the case of *LEC1* and *LEC2* also induces spontaneous SE (Parcy *et al.*, 1994; Lotan *et al.*, 1998; Stone *et al.*, 2001, 2008; Gazzarrini *et al.*, 2004; Braybrook *et al.*, 2006; Horstman *et al.*, 2017a). *LAFL* and *ABI* genes are regulated in part by larger, complex transcriptional and post-transcriptional feedback LAFL loops during seed development (Gazzarrini *et al.*, 2004; Zhang *et al.*, 2005; To *et al.*, 2006; Lepiniec *et al.*, 2018). Unlike *LEC1/2* overexpression, ectopic expression of *ABI* genes has not been reported to induce SE, but does confer seed maturation traits such as storage product accumulation (Parcy *et al.*, 1994; Reeves *et al.*, 2011).

*ABI4*, *ABI5*, and *LAFL* gene expression begins early in embryo development and decreases or becomes restricted to a subset of tissues in germinating seeds and seedlings (Brocard *et al.*, 2002; Kroj *et al.*, 2003; To *et al.*, 2006; Braybrook and Harada, 2008; Wang *et al.*, 2010; Wind *et al.*, 2013). Our data show that expression of *ABI3*, *ABI4*, *ABI5*, and other ABA signalling genes is maintained within the first 48 h of 2,4-D treatment (Fig. 2A), yet most known SE inducers or enhancers were either not differentially expressed or were down-regulated at the same time point (Supplementary Dataset S1). Three genes, *BBM*, *PLT1*, and *PLT2*, showed significant up-regulation after 2 d of 2,4-D treatment, but we demonstrated that at this time point, *BBM* is expressed in the root rather than the shoot, and that ectopic expression at the shoot apex, the site of somatic embryo initiation, occurs later, after 6 d of culture, following *LEC1* expression (Supplementary Fig. S5). These data suggest a two-step mechanism for SE induction in which 2,4-D first induces an ABA seed maturation response, followed by induction of embryo identity genes such as *LEC1* and *BBM* (Supplementary Fig. S13). 2,4-D and 2,4-D-induced maintenance of ABA-related gene expression during seed germination and beyond might be required to create a permissive transcriptional environment for expression of embryo identity genes such as *LEC1* and *BBM*.

Polycomb Repressive Complex 2 (PRC2) proteins regulate the transition from seed development to seed germination by repressing seed dormancy and embryo maturation traits in seedlings (Mozgova *et al.*, 2015). In Arabidopsis, shoots from 7-day-old *clf swn* seedlings occasionally make differentiated somatic embryos (Mozgová *et al.*, 2017). A combined wounding and 2,4-D treatment provides the additional competence for efficient somatic embryo induction in the shoot meristem of *clf swn* seedlings, but is not sufficient to induce SE from root tissues. ABA response appears to be limiting in *clf swn* roots, as addition of exogenous ABA to 2,4-D-treated roots is sufficient to induce SE. In this study, we found that endogenous ABA is limiting and required for efficient 2,4-D-induced SE from the shoot apex of wild-type mature embryo explants. However, exogenous ABA application does not induce SE from the explant root and actually inhibits SE from the shoot apex. This suggests that the pathways leading to 2,4-D-induced SE from mature embryos and from seedlings are different.

### Two mechanisms for SE from embryo explants

In Arabidopsis, SE can be induced from both immature bent cotyledon stage embryos and embryos from mature after-ripened seeds. SE efficiency is much higher in immature zygotic explants (~80%) than in embryo explants from mature after-ripened seed (~20%). The tissue competence for SE also differs between these two explants. In mature embryo explants, somatic embryos develop from the shoot apex, while in immature zygotic embryo explants somatic embryos develop from the cotyledon petioles and only a single somatic embryo develops from the shoot apex. Thus, overall and tissue competence for SE is gradually reduced during the late maturation phase (Gaj *et al.*, 2005; Wu *et al.*, 2019).

The *aba2*, *abi3*, *abi4*, and *abi5* mutants showed reduced SE from the shoot apex of mature geminating seeds, yet similar mutants show either normal or less severely reduced SE efficiency from the cotyledonary petioles of immature zygotic embryo explants (Gaj *et al.*, 2006). In wild-type immature zygotic embryo explants, somatic embryos develop from the petioles and shoot apex, but in *abi3-6* explants somatic embryos only develop from the petioles (Supplementary Fig. S9). This suggests two different mechanisms for 2,4-D-induced SE, one that operates in the cotyledonary petioles and one that operates in the shoot apex.

In immature Arabidopsis zygotic embryo explants, *LEC1/2* and *FUS3* are still relatively highly expressed in the cotyledons (Lotan *et al.*, 1998; Kroj *et al.*, 2003; To *et al.*, 2006). SE is severely compromised in *lec1*, *lec2*, and *fus3* immature zygotic embryos, and any embryos that develop, develop indirectly from callus rather than directly from the protoderm as in wild-type explants (Gaj *et al.*, 2005). Mature seeds show no or low *LEC1/2* and *FUS3* expression (Lotan *et al.*, 1998; Stone *et al.*, 2001; Lu *et al.*, 2010; Junker and Bäumlein, 2012), but *LEC1* expression can be induced after 4 d of 2,4-D treatment. Thus, the existence of a largely intact embryo identity programme in cotyledonary petioles might be sufficient to facilitate 2,4-D-induced SE from immature zygotic embryo cotyledons, even in the absence of individual *ABI* genes, while reinduction of a similar state is required for SE from the shoot apex of germinating embryos. These results are in line with studies showing that ectopic expression of the *LEC1*, *LEC2*, and *FUS3* seed maturation transcription factors can induce and/or enhance SE in different explants (Lowe *et al.*, 2003; Zhang *et al.*, 2014; Liu *et al.*, 2018).

Somatic embryo development from the shoot apex of germinating embryos relies on *ABI3*, *ABI4*, and *ABI5* (Fig. 5A). Little is known about the involvement of these genes in shoot meristem development, but roles for *ABI3*, *ABI4*, and *ABI5* in the auxin-dependent control of (lateral) root meristem size/number have been described (Brady *et al.*, 2003; Shkolnik-Inbar and Bar-Zvi, 2010; Yuan *et al.*, 2014; Ding *et al.*, 2015; Mu *et al.*, 2017). Only ABI3 has been shown to have a role in development of the shoot apex. *abi3* embryos show seedling-like characteristics, including premature activation of the shoot meristem and development of leaf primordia (Nambara *et al.*, 1995; Holdsworth *et al.*, 1999). *ABI3* also promotes vegetative shoot meristem quiescence in seedlings in response to ABA and dark (Rohde *et al.*, 1999). Meristems of dark-grown seedlings show ectopic *ABI3* expression and activation of a seed storage protein gene reporter (Rohde *et al.*, 1999), suggesting that meristem quiescence involves transdifferentiation to an embryogenic state. *ABI* gene expression is low/repressed in germinating embryos, allowing them to transition to vegetative growth (Lopez-Molina *et al.*, 2001, 2002; Wind *et al.*, 2013; Lepiniec *et al.*, 2018), but is maintained after 2,4-D treatment (Supplementary Fig. S4) . We propose that ectopic *ABI* expression in germinating embryos represses vegetative shoot differentiation, which provides the developmental framework required for 2,4-D-induced totipotent cell growth. In germinating embryos, 2,4-D represses vegetative shoot meristem development in favour of somatic embryo development, while (lateral root meristem) callus formation is induced on the abaxial surface of the cotyledons and in the basal region of the explant (Supplementary Fig. S1). *De novo* shoot organogenesis (pluripotency) in Arabidopsis has been shown to rely on 2,4-D-induced lateral root meristem formation from pericycle cells (Che *et al.*, 2007; Atta *et al.*, 2009; Sugimoto *et al.*, 2010). We showed that ABA biosynthesis mutants and mutants for positive ABA signalling components have reduced capacity for SE from the shoot apex, but increased callus formation in both the apical and basal regions of the explant (Figs 4C, F, 5C–E). ABA and ABI4 inhibit lateral root formation by reducing polar auxin transport (Shkolnik-Inbar and Bar-Zvi, 2010). Enhanced callus formation in ABA signalling mutants in 2,4-D-treated explants suggests that ABA and ABA signalling are required in these tissues to repress 2,4-D-induced lateral root formation. However, our mutant analyses show that enhanced ABA biosynthesis and signalling are not sufficient to induce SE in these tissues. Together, these data suggest that additional factors are limiting for 2,4-D,induced SE from the roots of mature embryo explants.

### A narrow window for somatic embryo induction from germinating seeds

*ABI3*, *ABI4*, and *ABI5* are required for efficient 2,4-D-induced SE from the shoot apex of germinating seeds (Fig. 5A). *ABI3/4/5* expression in germinating embryos declines during the first 2 d of seed germination, but is extended beyond this developmental window after treatment with 2,4-D (Supplementary Fig. S4). Our 2,4-D addition and removal experiments showed that this developmental window corresponds to the time frame in which the 2,4-D treatment is most effective for somatic embryo induction (Fig. 1Q, R). Treatment of germinating seeds with ABA within a short developmental window of 60 h after stratification can reinstate a seed ABA response and seed osmotolerance; thereafter ABA application induces a vegetative ABA response (Lopez-Molina *et al.*, 2001, 2002). ABI3 and ABI5 are both required to induce this developmental checkpoint. *ABI3* expression is up-regulated by ABA application within this developmental window, but not thereafter (Lopez-Molina *et al.*, 2002). *ABI3* acts upstream of *ABI5* to regulate *ABI5* expression, and ectopic expression of *ABI5* is sufficient to rescue the negative effect of the *abi3* mutant on this developmental checkpoint (Lopez-Molina *et al.*, 2002). Similarly, desiccation tolerance can be re-induced in a narrow developmental window during seed germination, and also relies on ABI3, ABI4, and ABI5 function (Maia *et al.*, 2014).

### A model for auxin–ABA interaction during induced cell totipotency

**Fig. 6.**
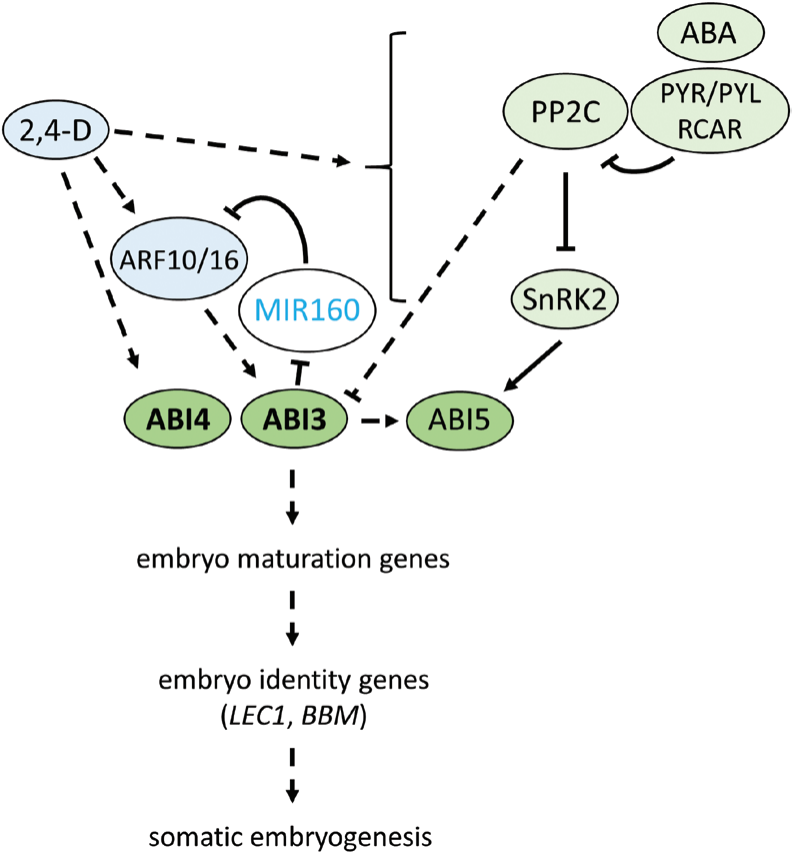
A proposed model for auxin and ABA interaction during somatic embryogenesis (SE) in Arabidopsis mature after-ripened embryos. Auxin treatment induces expression of ABA signalling genes and maintains expression of the downstream *ABI3/4/5* transcription factor genes. ABA signalling and ABI5 transcription factors are required for efficient SE from germinating seeds, while ABI3 and ABI4 (bold) are essential for SE. ABA signals through the upstream components, including the receptors and the PP2Cs, to indirectly up-regulate ABI3 (Lopez-Molina *et al.*, 2002), while core ABA signalling promotes ABI5 phosphorylation (Fujii *et al.*, 2009; Melcher *et al.*, 2009; Yin *et al.*, 2009). ABI3 also regulates *ARF10* and *ARF16* expression through *MIR160*, which post-transcriptionally regulates *ARF10* and *ARF16* levels (Tian *et al.*, 2020b). Dashed lines indicate indirect transcriptional regulation, while black solid lines indicate known protein-level regulation.

We propose a model (Fig. 6) in which 2,4-D promotes an ABI-mediated transcriptional cascade in germinating seeds leading to repression of vegetative meristem development in favour of somatic embryo induction. This pathway is dependent on ARF10/16 and on signalling through the core ABA signalling pathway (RCARs/PYR1/PYLs–PP2Cs–SnRK2s), as reduced/enhanced ABA signalling negatively/positively affects 2,4-D-induced SE, respectively. ABA positively regulates *ABI3/4/5* expression (Lopez-Molina *et al.*, 2001, 2002; Arroyo *et al.*, 2003; Shkolnik-Inbar and Bar-Zvi, 2010), ABI3/4/5 protein stability/accumulation (Lopez-Molina *et al.*, 2001, 2002; Shu *et al.*, 2016a), and ABI5 protein phosphorylation (Lopez-Molina *et al.*, 2001; Fujii *et al.*, 2009; Melcher *et al.*, 2009; Yin *et al.*, 2009). ABI3 and ABI4 play a larger role in SE than ABI5, as increased signalling through the ABA receptor can partially restore SE efficiency in the *abi5* mutant, but not in the *abi3* and *abi4* mutants. Auxin and ABA might interact synergistically through an ARF10/ARF16–*ABI3* expression module to regulate SE, as was shown for seed germination (Liu *et al.*, 2013). Together, this model provides a new framework for identifying additional, intersecting plant totipotency pathways, and for directing efficient SE in systems that make use of mature seed explants (Wu *et al.*, 2019).

## Supplementary data

The following supplementary data are available at *JXB* online.

Table S1. Plant materials.

Table S2. Primers used in this study.

Fig. S1. Development of the root hypocotyl region in 2,4-D-treated explants.

Fig. S2. GO analysis of 2,4-D differentially regulated genes.

Fig. S3. Statistically significant differentially regulated auxin pathway genes.

Fig. S4. qRT–PCR validation of differentially expressed ABA-related genes in SE culture.

Fig. S5. 2,4-D treatment induces ectopic *BBM:BBM-GUS* expression post-germination.

Fig. S6. The effect of ABA application on SE in ABA receptor mutant explants.

Fig. S7. *35S:PYL10* and *35S:ABI3* overexpression lines.

Fig. S8. Effect of *abi3* weak alleles on 2,4-D-induced somatic embryogenesis.

Fig. S9. Effect of the *abi3-6* allele on 2,4-D-induced somatic embryogenesis from immature zygotic embryo explants.

Fig. S10. Effect of ABA application on SE efficiency in *abi3*, *abi4*, and *abi5-7* explants.

Fig. S11. 2,4-D treatment maintains *ABI3* and *ABI4* expression post-germination.

Fig. S12. ARF10 and ARF16 are required for 2,4-D-induced SE.

Dataset S1. Microarray data (all data, ABA-related genes, auxin-related genes, seed maturation-related genes, GO analysis of 2,4-D-induced DEGs, somatic embryogenesis inducer and enhancer genes).

erab306_suppl_Supplementary_Materials

erab306_suppl_Supplementary_Data_Set_1

## Data Availability

All data supporting the findings of this study are available within the paper and within its supplementary data published online.

## Abbreviations

ABA: abscisic acid
2,4-D: 2,4-dichlorophenoxyacetic acid
SE: somatic embryogenesis
