# Supplementary material for: ABA signalling promotes cell totipotency in the shoot apex of germinating embryos
Source: J Exp Bot. 2021 Jun 27;72(18):6418–36. doi: 10.1093/jxb/erab306 (PMC8483786; doi:10.1093/jxb/erab306)
Supplement: erab306_suppl_Supplementary_Materials [file erab306_suppl_supplementary_materials.pdf]

SUPPLEMENTARY DATA

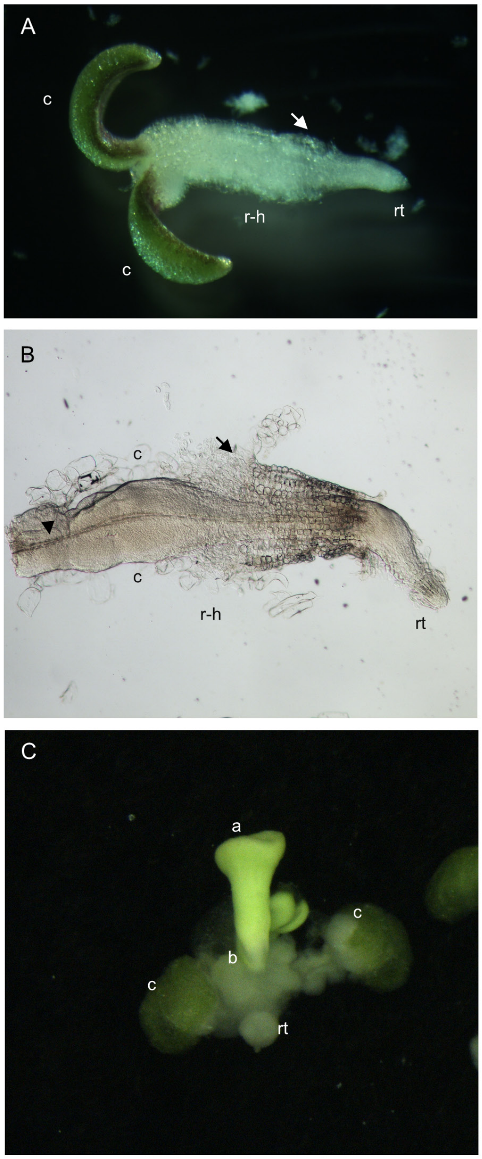

Supplementary Fig. S1. Development of the root hypocotyl region in 2,4-D-treated explants. **A**, Four-day-old WT Col-0 explant from somatic embryo culture. c, cotyledon; r-h, root-hypocotyl region; rt, root tip. Arrow, the loose outer layer of the root-hypocotyl region. **B**, Close up of the root-hypoctoyl region of an eight-day-old WT Col-0 explant. C, callus; r-h, root-hypocotyl region; rt, root tip. Arrowhead, vascular tissue cylinder, arrow, detached epidermal/subepidermal cells. **C**, Embryogenic explant (14 d), with a bipolar embryo. c, cotyledon; rt, root tip; a and b indicate the apical pole (the future shoot) and basal pole (the future root), respectively. The images are light micrographs.

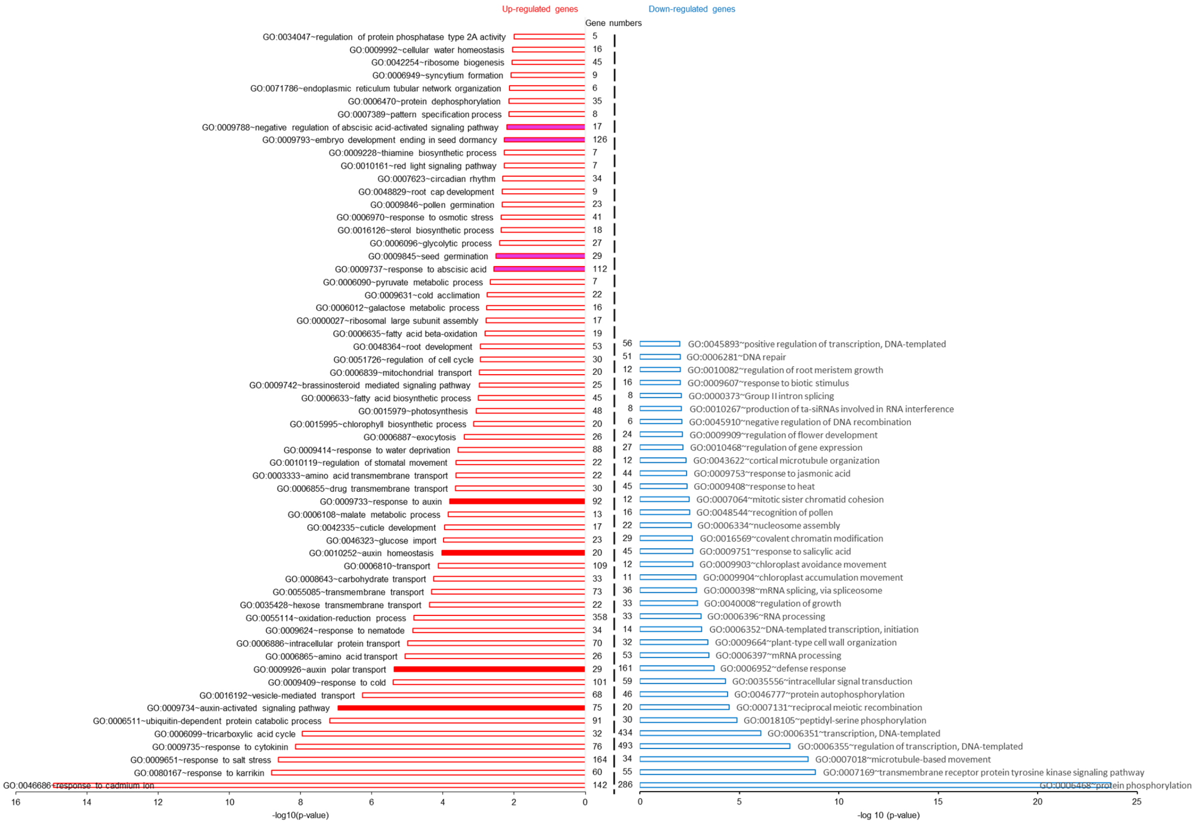

Supplementary Fig. S2. GO analysis of 2,4-D differentially-regulated genes. GO analysis of 2,4-D significantly differentially-regulated genes (log2 fold change > 0.5 or < -0.5) responsive to 2,4-D treatment. The top GO terms (with *p*-values < 0.01) are shown here with the *p*-value at the end of each bar. The GO analysis was performed using DAVID (https://david.ncifcrf.gov/summary.jsp) with EASE 0.05 (a cut-off value of *p* < 0.05).

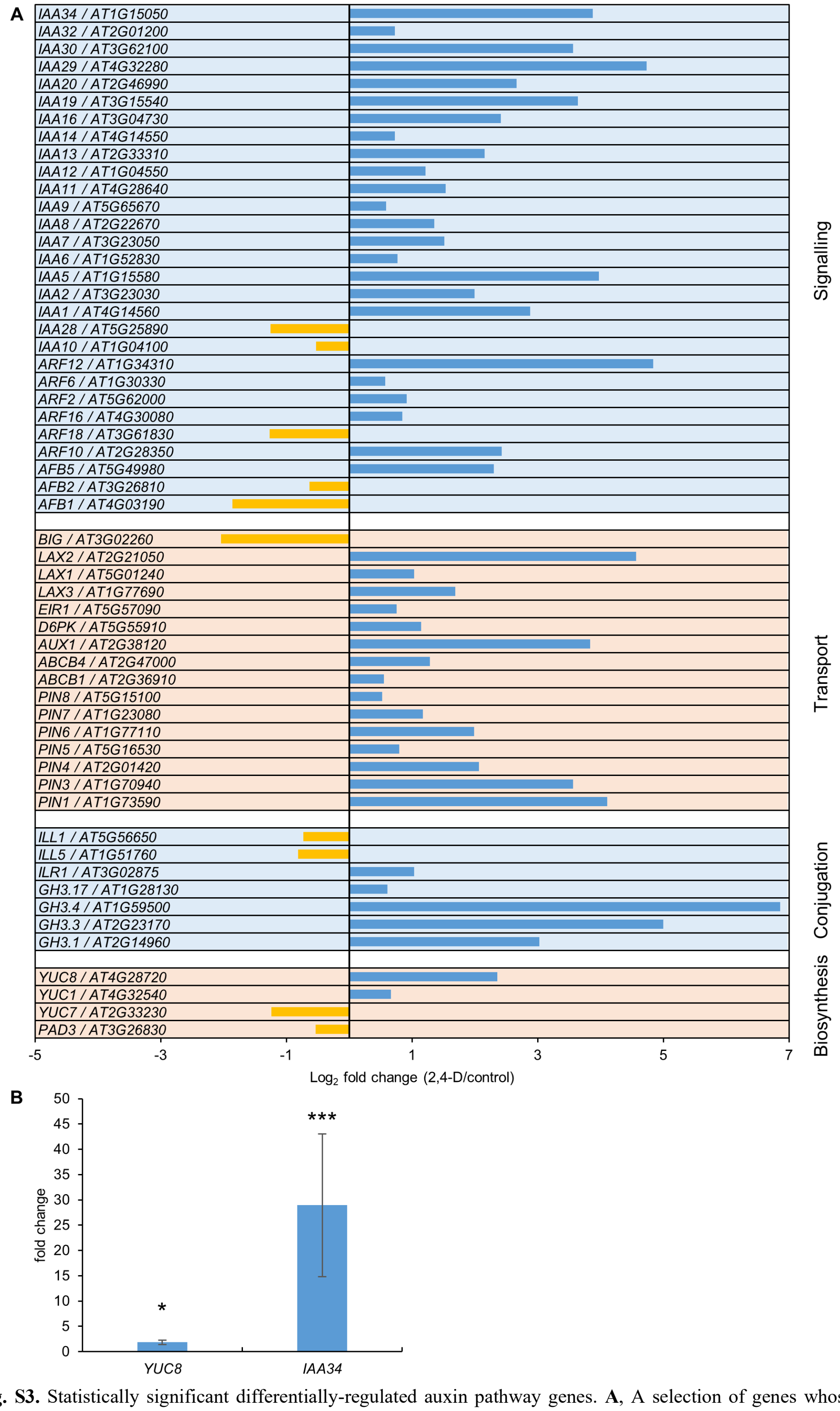

Supplementary Fig. S3. Statistically significant differentially-regulated auxin pathway genes. **A**, A selection of genes whose expression was altered in germinating seeds in response to 2,4-D treatment is shown. The gene name and arabidopsis gene identifier (AGI), as well as the $\log_2$ fold change in expression between 2,4-D-treated seeds and control seeds are shown for each gene. Genes were grouped per functional category. The complete data set can be found in Supplemental Data Set 1. **B**, qRT-PCR validation of differentially-expressed auxin-related genes. Relative expression ($2^{-\Delta\Delta C_T}$) was determined by qRT-PCR in two-day-old explants cultured in the presence or absence (calibrator) of 2,4-D. Statistically significant differences between 2,4-D treatment and the control were calculated using a two-tailed Student's t-test (*, $p < 0.05$; ***, $p < 0.001$). Error bars represent the standard deviation of three biological replicates.

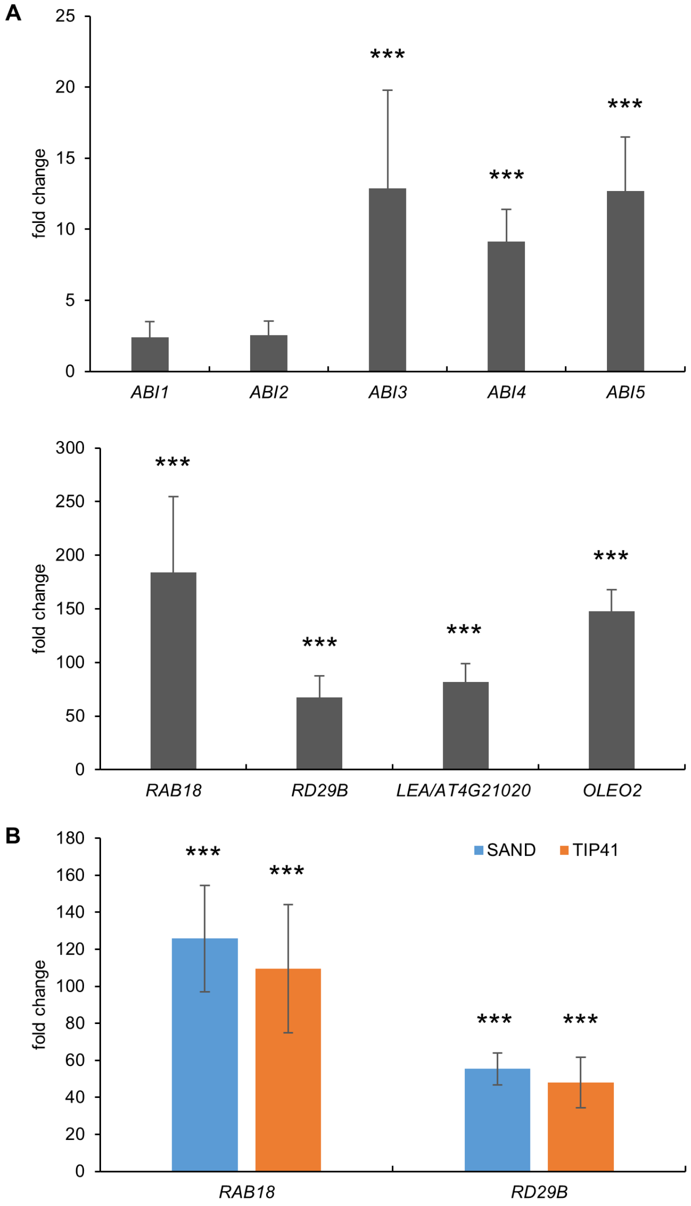

Supplementary Fig. S4. qRT-PCR validation of differentially-expressed ABA-related genes in SE culture. **A**, Relative expression ($2^{-\Delta\Delta C_T}$) was determined in two-day-old explants that were cultured in the presence or absence (calibrator) of 2,4-D. *SAND* was used as the reference gene. **B**, Validation of the qRT-qPCR results in (A) for *RAB18* in the higher expression range and *RD29B* in the lower expression range using two reference genes (*SAND* and *TIP41*). Statistically significant differences between 2,4-D treatment and the control were calculated using a two-tailed Student's t-test (***, $p < 0.001$). Error bars represent the standard deviation of three biological replicates.

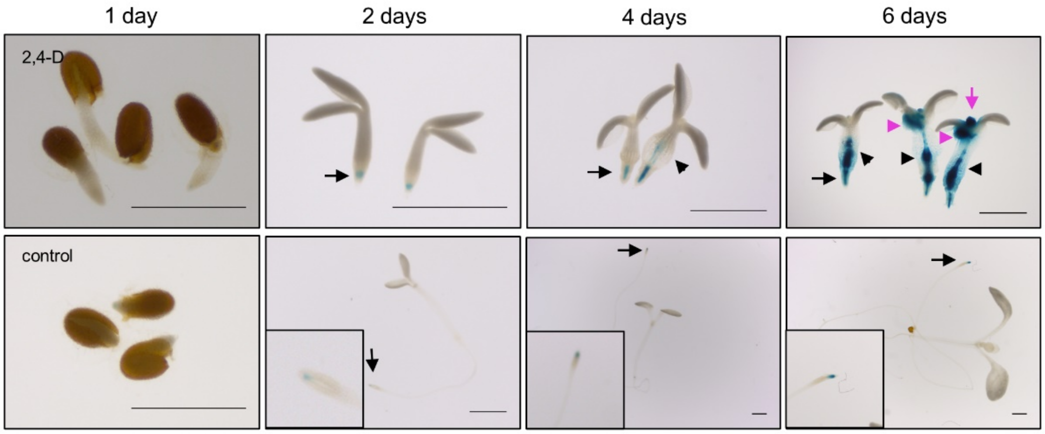

Supplementary Fig. S5. 2,4-D treatment induces ectopic *BBM:BBM-GUS* expression post-germination. *BBM:BBM-GUS* expression in somatic embryo cultures in the presence or absence (control) of 2,4-D. The insets are magnifications of the seedling root tip. The days of culture are indicated above the panels. The images are light micrographs. Black arrow, root tip expression; black arrowhead, hypocotyl expression; pink arrow, SAM expression; pink arrowhead, expression from callus on the petiole. Scale bars, 1mm.

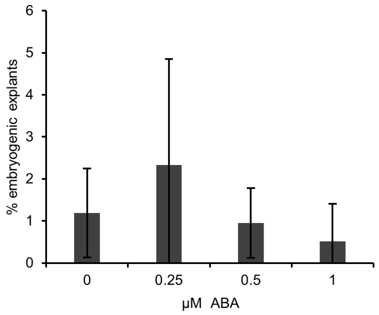

Supplementary Fig. S6. The effect of ABA application on SE in ABA receptor mutant explants. Effect of exogenous ABA application on 2,4-D-induced SE from the *pyl112458* ABA receptor mutant. The Col-0 control was previously reported by Wu *et al.* (2019). Statistically significant differences in SE efficiency between ABA and the mock treatment were calculated using a two-tailed Student's t-test. No statistically significant difference was found. Error bars represent the standard deviation of three technical replicates.

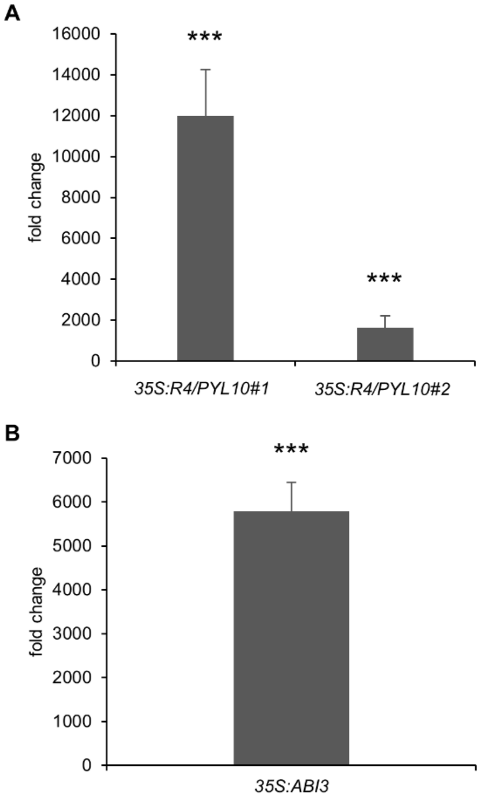

Supplementary Fig. S7. *35S:PYL10* and *35S:ABI3* overexpression lines. Elevated *PYL10* (A) and *ABI3* (B) gene expression was confirmed by qRT-PCR ($2^{-\Delta\Delta C_T}$) by comparing seven-day-old *35S:PYL10* or *35S:ABI3* seedlings with seven-day-old WT Col-0 seedlings (calibrator). Statistically significant differences between overexpression lines and the control were calculated using a two-tailed Student's t-test (***, $p < 0.001$). Error bars represent the standard deviation of three technical replicates.

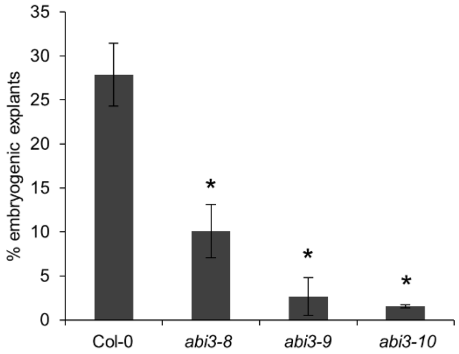

Supplementary Fig. S8. Effect of *abi3* weak alleles on 2,4-D-induced somatic embryogenesis. Statistically significant differences in SE efficiency between WT Col-0 and the indicated mutant explants were calculated using a two-tailed Student's t-test (*, $p < 0.05$). Error bars represent the standard deviation of three technical replicates.

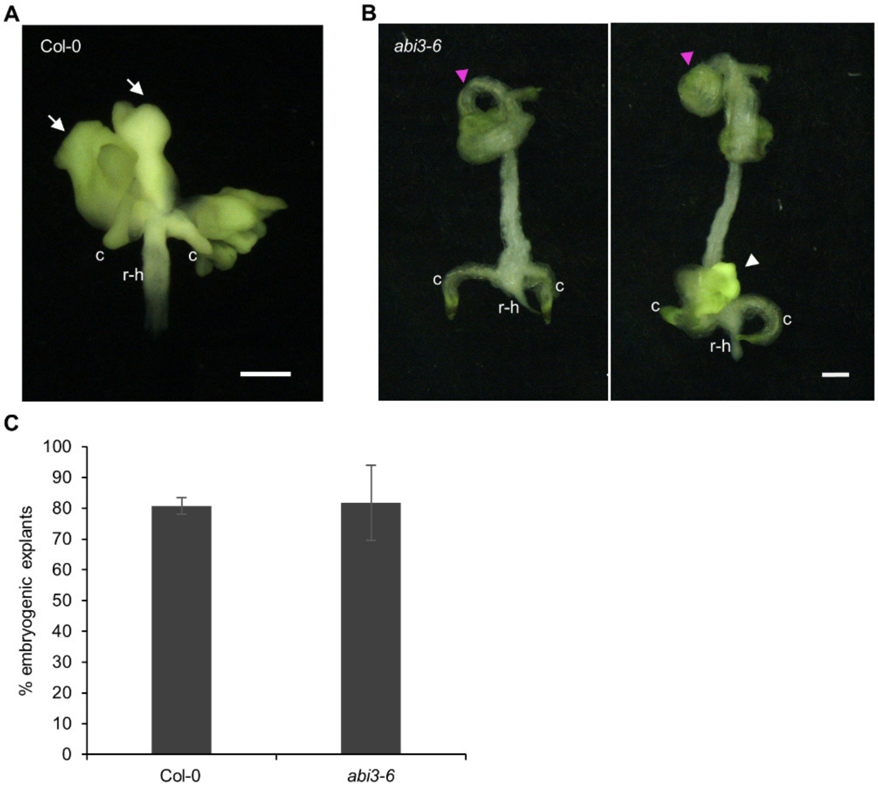

Supplementary Fig. S9. Effect of the *abi3-6* allele on 2,4-D-induced somatic embryogenesis from immature zygotic embryo explants. **A-B**, Representative embryogenic explants from WT Col-0 (A) and *abi3-6* (B) 14 days after the start of culture. The images are light micrographs. c, cotyledon; r-h, root hypocotyl region; white arrow, somatic embryo; pink arrowhead, shoot-like structure; white arrowhead, embryogenic tissues. Left panel, non-embryogenic explant; right panel, embryogenic explant. Scale bars, 1 mm. **C**, SE efficiency of WT and *abi3*-6 immature zygotic embryo explants. No statistically significant differences in SE efficiency were observed between WT and *abi3*-6 explants (two-tailed Student's t-test). Error bars represent the standard deviation of three technical replicates.

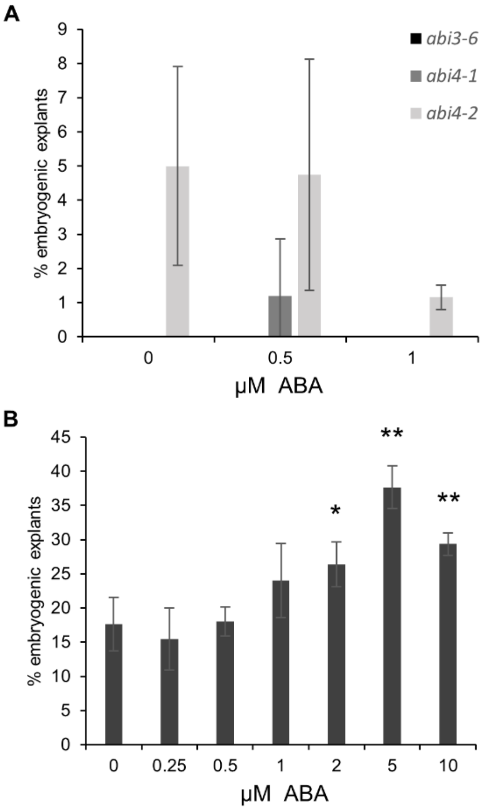

Supplementary Fig. S10. Effect of ABA application on SE efficiency in *abi3*, *abi4* and *abi5-7* explants. **A**, Effect of exogenous ABA application on 2,4-D induced SE from the *abi3-6*, *abi4-1* or *abi4-2* mutants. No statistically significant differences in SE efficiency were observed between treatments with and without ABA (two-tailed Student's t-test). Error bars represent the standard deviation of three technical replicates. **B**, Effect of exogenous ABA application on 2,4-D induced SE from the *abi5-7* mutant. Statistically significant differences in SE efficiency between ABA and mock treatments were calculated using a two-tailed Student's t-test (*$p < 0.05$; **, $p < 0.01$). Error bars represent the standard deviation of three technical replicates.

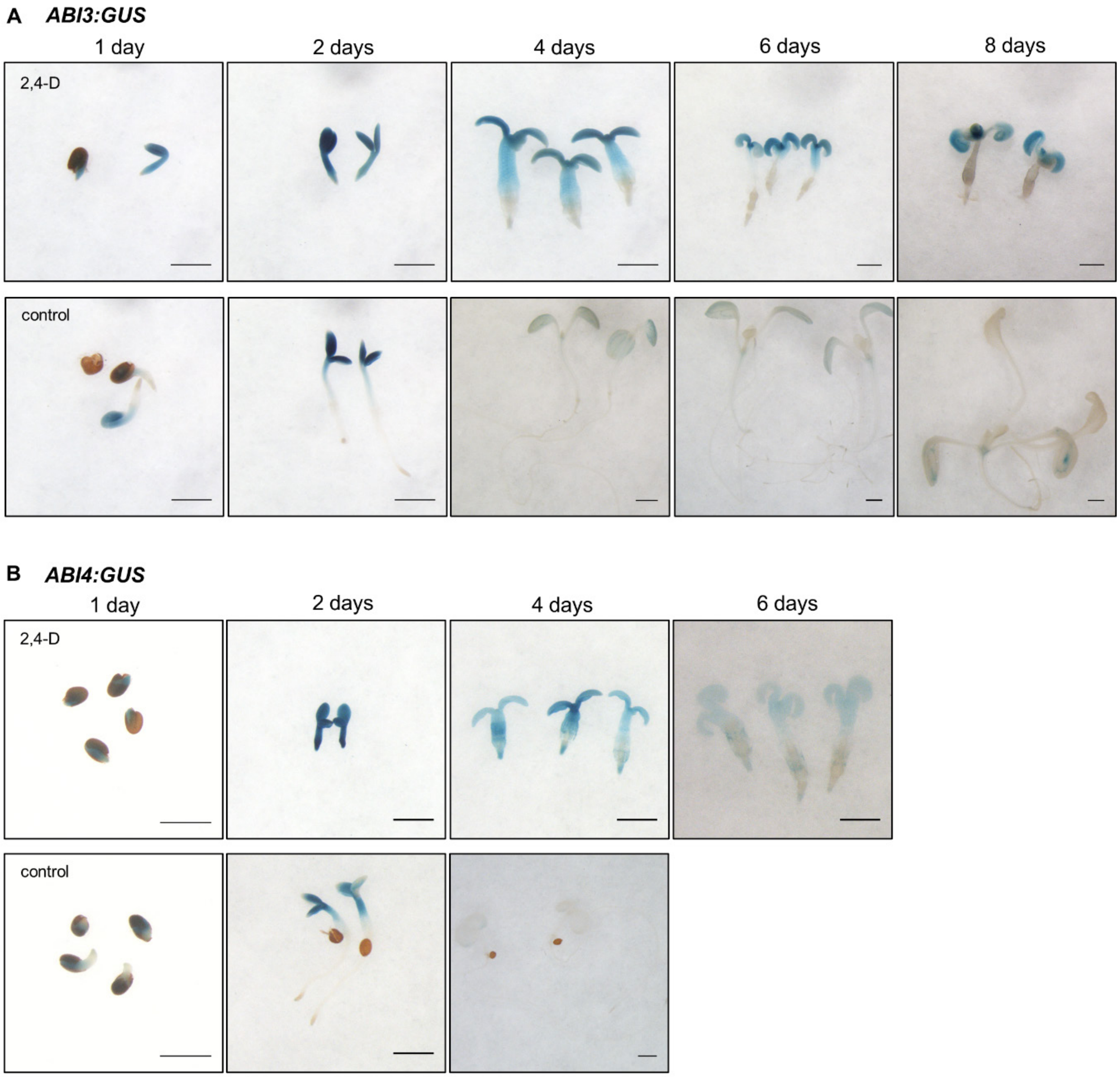

Supplementary Fig. S11. 2,4-D treatment maintains *ABI3* and *ABI4* expression post-germination. *ABI3:GUS* (A) and *ABI4:GUS* (B) expression in time after culture in the presence or absence (control) of 2,4-D. The days of culture are indicated above the panels. The images are light micrographs. Scale bars, 1mm.

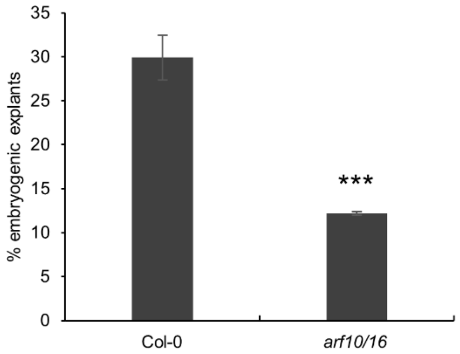

Supplementary Fig. S12. ARF10 and ARF16 are required for 2,4-D-induced SE. Effect of the *arf10/16* double mutant on 2,4-D-induced SE. Statistically significant differences in SE efficiency between WT Col-0 and mutant explants was calculated using a two-tailed Student's t-test (***, $p < 0.001$). Error bars represent the standard deviation of three technical replicates.

Supplementary Table S1. Plant materials.

Arabidopsis lines	Source	Identifier
aba2-1	Leon-Kloosterziel et al., 1996	N/A
cyp707a2-1	Kushiro et al., 2004	N/A
pyl10^{CR}	This study, 264 bp deletion (after ATG, from +23 to + 286)	N/A
pyl8-1 pyl10^{CR}	This study	N/A
pyl8-1 pyl9	Xing et al., 2016	N/A
pyl8-1 pyl9 pyl10^{CR}	This study	N/A
pyl112458	Gonzalez-Guzman et al., 2012	N/A
pyl112458379101112	Zhao et al., 2018	N/A
hab1-1	Saez et al., 2004	Salk_002104C
ahg3	This study	Salk_009863C
abi1-2 hab1-1	Rubio et al., 2009	N/A
Snrk2.2 snrk2.3 snrk2.6 triple	Fujii and Zhu, 2009	N/A
abi3-6	Nambara et al., 1994	N/A
abi3-8	Nambara et al., 2002	N/A
abi3-9	Nambara et al., 2002	N/A
abi3-10	Nambara et al., 2002	N/A
abi4-1	Finkelstein et al., 1998	N/A
abi4-2	Quesada et al., 2000	N/A
abi4-3	Dijkwel et al., 1996, 1997	N/A
abi5-7	Nambara et al., 2002	N/A
arf10/16	Wang et al., 2005	N/A
35S:R4/PYL10 #1	This study	N/A
35S:R4/PYL10 #2	This study	N/A
35S:R12/PYL1	Yang et al., 2016	N/A
35S:ABI1	Wang et al., 2018	N/A
35S:ABI2	Wang et al., 2018	N/A
35S:ABI3	This study	N/A
35S:ABI4	Shu et al., 2013	N/A
35S:ABI5	Brocard et al., 2002	N/A
LEC1:LEC1-GFP	Li et al., 2014	N/A
ABI3:GUS	Ryu et al., 2014	N/A
ABI4:GUS	Söderman et al., 2000	N/A
BBM:BBM-GFP-GUS	This study	N/A

N/A, not applicable

Supplementary Table S2. Primers used in this study.

**Cloning**		
PYL10	Fw	GGGGACAAGTTTGTACAAAAAAGCAGGCTCGATGAACGGTGACGAAACAAAG
	Rv	GGGGACCACTTTGTACAAGAAAGCTGGGTCTCATATCTTCTTCTCCATAGATTC
ABI3	Fw	TGTTTCATTTCCACTTCAACG
	Rv	GTTTCTTTTTGTCTCTGTTTTTCATTT
BBM	Fw	GGGGACAAGTTTGTACAAAAAAGCAGGCTCAGAAAGCTTACGATTACAGAGACCA
	Rv	GGGGACCACTTTGTACAAGAAAGCTGGGTGAAGTGTCGTTCCAAACTGAAAACG
OLE1:OLE1-RFP	Fw	AGTCTCTAGAACTAAATGGAGCAACCTACTGTTTTTG
	Rv	AGTCACTAGTAATGTCGCGGAACAAATTTTAAAAC
**CRISPR-Cas9**		
gRNA 1	Fw	TGTGGTCTCAATTGCGGTGACGAAACAAAGAAGGGTTTTAGAGCTAGAAATAGCAAG
gRNA 2	Fw	TGTGGTCTCAATTGCAAAGCTTACGAGATGCAGGTTTTAGAGCTAGAAATAGCAAG
gRNA 3	Fw	TGTGGTCTCAATTGAAAAGGTGTGGTCAATTGTGGTTTTAGAGCTAGAAATAGCAAG
gRNA 4	Fw	TGTGGTCTCAATTGGTACAAGGTAAGAAGCTGGGTTTTAGAGCTAGAAATAGCAAG
CRISPR Universal	Rv	TGTGGTCTCAAGCGTAATGCCAACTTTGTAC
**Genotyping**		
pyl8-1	LP	AGAGAGTGGAACCCCATGATC
	RP	TTCTTCTTCTTCCTTCATGCG
pyl9	LP	TTCACTTCAATGCCCTTGTTC
	RP	TAGGTCCCCAAAACGTCATAC
*pyl10^CR^*	Fw	TCCTTTAAATAGCTGCACACTGGT
	Rv	TCATATCTTCTTCTCCATAGATTC
hab1-1	LP	ACAATGGCTTGTAGGTTGCTG
	RP	CGAAAACTCGAAACTTACCCC
ahg3	LP	GTAATCACGGAACGGATTCAC
	LP	CGGTTCAGTCTCTCCAACAAC
LBb1.3	BP	ATTTTGCCGATTTCGGAAC
**qRT-PCR**		
SAND	Fw	AACTCTATGCAGCATTTGATCCACT
	Rv	TGATTGCATATCTTTATCGCCATC
TIP41	Fw	CACCATGGACCAGTATGAAAAAGTTGAAAAG
	Rv	TCATGGTACGAACCCAATGTCC
YUC8	Fw	TGCGGTTGGGTTTACGAGGAAAG
	Rv	GCGATCTTAACCGCGTCCATTG
IAA34	Fw	GTTTGGTGGTAGGTCGCAAG
	Rv	ATCCCCAGCATTTCTCCACA
ABI3	Fw	GGCAGGGATGGAAACCAGAAAAGA
	Rv	GGCAAAACGATCCTTCCGAGGTTA
ABI4	Fw	GGGCAGGAACAAGGAGGAAGTG
	Rv	ACGGCGGTGGATGAGTTATTGAT
ABI5	Fw	CAGCTGCAGGTTCACATTCTG
	Rv	CACCCTCGCCTCCATTGTTAT
RAB18	Fw	TCGGTCGTTGTATTGTGCTTTTT
	Rv	CCAGATGCTCATTACACACTCATG
ABI1	Fw	TGAGATGGCAAGGAAGCGGATTCT
	Rv	GGCTTCAAATCAACCACCACCACA
ABI2	Fw	ACACGTGGCAAGAGAAGTGGAAGA
	Rv	CCGCAATTCGCGACAAAGATGTGA
LEA	Fw	CGGCCATGCAACTAACAAGAACC
	Rv	GAGACTTTCCGGGAAGAAGCAGC
OLEO2	Fw	GCCGAGGTCAAGGTCAGTATGAAGG
	Rv	GGGTACTAGATGGGCCACTTTCAGG

RD29B	Fw	GGCGGGCAAAGCGAG
	Rv	TGCCCGTAAGCAGTAACAGATC

Note: Forward primers for cloning gRNAs are shown with the target sequence in red.

Fw, Forward primer; Rv, Reverse primer. LP, Left genomic primer; RP, Right genomic primer; BP, T-DNA border primer.
